# Maximizing tree harvesting benefit from forests under insect infestation disturbances

**DOI:** 10.1371/journal.pone.0200575

**Published:** 2018-08-02

**Authors:** Maria C. A. Leite, Benito Chen-Charpentier, Folashade B. Agusto

**Affiliations:** 1 Mathematics and Statistics, University of South Florida St. Petersburg, St. Petersburg, Florida, United States of America; 2 Department of Mathematics, University of Texas, Arlington, Texas, United States of America; 3 Department of Ecology and Evolutionary Biology, University of Kansas, Lawrence, Kansas, United States of America; University of Waterloo, CANADA

## Abstract

Mathematical modeling has been recognized as an important tool to advance the understanding of the synergetic effect of coupled disturbances (stressors) on the forest population dynamics. Nonetheless, most of the modeling done on disturbances focus on individual disturbance agents and the modeling research on disturbances interactions uses predominantly descriptive statistical processes. This state of art points to the need for continuing modeling efforts not only for addressing the link among multiple disturbances but also for incorporating disturbance processes. In this paper, we present an age-structured forest-beetle mechanistic model with tree harvesting. We investigate three scenarios involving the beetles equilibrium states (no beetles, beetles in endemic and epidemic states). Optimal control theory was applied to study three different benefit functions involving healthy and dead trees. The numerical simulations show that maintaining the beetle infestation at endemic level instead of eliminating all the beetles is sufficient to ensure the forest has trees with all ages. Furthermore, the numerical simulations shows that the harvesting benefit decreases as the number of beetles increases in all cases except when the benefit functional includes a cost (ecological and harvest implementation) and the value of wood is equal across all trees (healthy harvested trees, trees killed by beetles, and trees that die naturally).

## Introduction

Forests are a vital ecological and economical resources and forest management is a complex problem. Mainly, because of the diversity of the landscape, the inter-relationships between the different components of the forest, and the wide ranges of values associated with natural resources. The level of complexity associated with the sustainable management of forest increases due to distinct anthropological disturbances and the unpredictability of natural disturbances occurring in forests. For example, currently, in the face of recurring insect outbreaks forest planning and regenerative measures have become more difficult [1–7]. This situation has prompted professional foresters, researchers, landowners, and concerned citizens to reassess forest management practices and to consider less-common silvicultural management techniques [3, 4, 7–9]. However, in order to develop or select efficient management strategies that fit the current situation, it is critical to understand the impacts of the different factors (such as age distributions, insect outbreaks, fire size and frequency, harvesting, and drought), to increase the knowledge of the interactions among them, and to understand their main effects on the forest dynamics. This understanding is particularly pertinent in the context of beetle infestations and harvesting since they constitute two major forest disturbances in North America [10–12].

Dynamics of beetle population and its impact on forest have inspired a vast body of work; however, complete beetle or beetle-disease population dynamics or beetle-disease prognostic models are currently lacking [13, 14]. Furthermore the current understanding of the complex interactions among disturbances at a process level is still limited [8, 13, 15].

Mathematical modeling has been recognized as an important tool to advance the understanding of the synergistic effect of coupled disturbances on the forest population dynamics [15–18]. Nonetheless, most of the modelling work focus on individual disturbance agents and the existing modelling research on disturbances interactions uses predominantly descriptive statistical processes [13, 15]. This state of art points to the need for continuing modeling efforts not only for addressing the link among multiple disturbances but also for incorporating disturbance [15]. On the other hand, optimal control theory provides an analytical starting point for searching optimal harvesting strategies and to identify some options for further investigation.

Optimal control problem involving harvesting of age- and size-structured single and multi-species has been widely studied in the literature (e.g., [4, 19–23]), especially for forests and fish populations, either generally or/and applied to forest and fish of specific regions and populations.

Despite the vast body of literature on dynamics of beetle populations and optimal control in harvesting context, there are only a few studies that aim to bridge both of them so as to gain insights on the potential new effects of coupling harvesting and insect outbreak disturbances on forest dynamics and optimal logging strategies. For example, in [24] a numerical simulation approach was used to study an optimization problem with the purpose of studying the effects of insect outbreaks on the financial performance of Dahurian larch. An individual-tree model is presented where the insect infestation is only considered to be at epidemic state. The severity of the epidemic is simulated as random events. The work of [25] studies an optimal control problem of a bioeconomic model of timber harvesting that includes interaction between mountain pine beetle and lodgepole pine forest with disaggregated size structure. The model is discrete and does not consider the effects of forests on the pest.

On the other hand, the influence of disturbances (including insect infestations) on the age-structure dynamics of forest systems *per se* have not been studied extensively [1, 8, 9, 13]. In particular, there are not many studies within the context of mathematical ecology. Two interesting works addressing age-structure in forests can be found in [26] and [27]. In [26] an age-structured forest demographic model incorporating temperature-dependent mountain pine beetle infestations was developed for characterizing outbreak infestation and predicting potential severity of future outbreaks that reflect the effects of changing climate. The tree population is structured into distinct age classes leading to a system of nonlinear difference equations. The authors also include spatial variability by coupling compartmental distinct stands. The work in [27] studies how host selection and mass attack influence the dynamics of beetle outbreaks. The tree population is structured by distinct levels of both the vigor and size (bark area) of a tree within a stand. The model was simplified by assuming that beetles distribute themselves only according to host vigor and not according to host tree bark area.

The resulting vigor-structured model for mountain pine beetle outbreak dynamics consists of a system of nonstandard nonlinear integrodifference equations. Under certain assumptions the model was reduced to a delayed discrete-time dynamical system. The authors also extended the model via a stochastic formulation. Both works include a fair amount of detail concerning mountain pine beetle population dynamics. However, the addressed problem differs from the one we are studying in this paper, which is the study of the impact of coupling harvesting and insect outbreaks on the forest structure as well as using optimal control theory for exploring harvesting strategies that maximize harvest benefit in the presence of distinct beetle population levels.

In a recent paper [5] we developed an age-structured mathematical model for the growth of trees subject to two disturbances: beetle infestation and harvesting. The model is deterministic and does not include the feedback of the tree population on the beetle dynamics. We consider three insect infestation states: no beetles, endemic state, and epidemic state and investigated the optimization of the harvesting benefit over two distinct logging scenarios: cutting all trees older than a given age and cutting a fixed proportion of trees older than a given age. This model as well as the model formulated in [25] may be inadequate if the changes in the forest structure have an effect on pest dynamics as the latter should somehow be connected to the density of available hosts and some of their characteristic [26–28]. The work in [24] consider only beetle population in its epidemic state, thus there is no comparison of results across multiple levels of forest infestation (absence of beetles, endemic state, epidemic state).

To address the shortcoming of those models in this paper we extend the mathematical models in [5] by including the feedback of tree population dynamics on beetle population dynamics (section: “Beetle infestation in an age-structured tree population model”). This feedback is introduced by explicitly modeling the influence of the age of trees on the beetle population dynamics. In addition to studying the harvest benefit considered in [5], that is, the total number of trees harvested, we study two more realistic harvest benefit functions: the total amount of wood harvested and total benefit. We investigate three distinct optimal control problems, which aim to maximize each of the three benefit functions from harvesting of trees in forests with beetle infestations (section: “Optimal control problem”). Since there is no full generalization of Pontryagin’s maximum principle for the control of partial differential equation (PDE) we have to prove the existence of an optimal control and establish the necessary conditions for optimality (section: “Optimal control existence and characterization”). The methods used follow those found in the work of [29–31]. The optimal control problem addressed in [30] is similar to the ones we studied in this work. Once we have proved the existence and uniqueness of an optimal control and characterized the optimal control for each of the three problems, we use numerical methods to simulate some biological scenarios to illustrate our results. These are presented in the section: “Numerical simulations” and section: “Numerical results”. In section: “Discussion of results”, we present a brief discussion of the findings.

## Beetle infestation in an age-structured tree population model

The mathematical model adopted in this paper, introduces an explicit linkage between the beetle population dynamics and an age-structured forest population dynamics subject to tree harvesting. The formulation builds upon the approach in [5] by adding the explicit dependence of the carrying capacity of the beetle population on the age of trees. Thus, the model presented here includes feedback of trees dynamics on the beetle population dynamics and vice-versa. The interactions between insects and trees are modeled similarly as age-structured forest-disturbances models that use Lotka-Mckendrick partial differential equations (PDE) predator-prey type models (e.g., [30, 32, 33]). Most of these models assume that only one species, either the predators or the preys, are structured by certain individual variability. That is, classify either prey or predator individuals by some (continuous) internal variables as age, size, vigor, energy, reserves or whatever variable(s) that reflects it and has an actual influence on the population dynamics [33, 34].

In this paper we assume the dynamics of trees (the prey) are described by a linear Lotka-McKendrick model [35] structured by age of trees, which is an idea similar to the one used in [26]. But in the latter the authors used a compartmental (classes) structure while we use continuous age structure formulation. The choice of age for tree population structure was also motivated by the fact that most of the bibliography for general harvesting models (see [29, 36–38] and references therein) use this approach. The size-structure could also be used but there is some evidence that size and age of a tree can be related through the diameter at breast height (DBH) [26]. Other variable could be used to structure the tree population such as vigor, or bark area (as in [27]), or other characteristics that beetles may cue on (as suggested in [27]).

Another model framework that could be considered is the Mitra-Wan model [39, 40] formulation: either the classic formulation as a discrete model where all trees are planted young and the tree population is kept constant [4, 41]; or the continuous-time version recently proposed by Fabbri *et al*. [42], which assumes that the saplings of age zero at time zero coincides with the total amount of trees (of different ages) cut at time *t*, which is equivalent to the assumptions associated to the discrete-time Mitra-Wan model. Under Lotka-McKendrick model framework, which we follow in this work, the new trees in the system, that is, the trees of age zero at a given time follow a function that depends on their age. This assumption reflects the fact that the trees produce fruit/seeds that depends on the age of each tree.

Also, the model could be constructed using ideas introduced in [26] where the tree population is structured by age classes and the infestation by beetles is given by a temperature-driven Ricker-type model. The nonstandard nonlinear integrodifference equations model in [27], could be adapted to incorporate harvesting stressor (disturbance). However, both models incorporate a significant biological details on the dynamics of the insect infestation. While more complex models are arguably more realistic any attempt in this direction would be mathematically challenging when addressing the optimal control study. Additionally, would require a multitude of parameters and, necessarily, data to validate it. Given the nature of the phenomena at hand, these requirements may be very difficult to satisfy. Therefore, although we couldn’t identify data to validate our simplified models either, the approach of using simpler models may have advantages by providing theoretical insights.

The dynamics of the beetle population are described by an extension of the model with age-independent carry capacity are given in [5]. The state variables are *B*(*a*, *t*) and *V*(*a*, *t*), representing the average number of beetles per tree of age *a* and the age density of susceptible trees, respectively, both at time *t*.

One of model underlying assumptions is that the age of trees is finite. This is an assumption that has been used by others in the context of age-structured models [36, 43]. We believe this assumption is reasonable since the life span of a tree can be very long but eventually the tree will die [44, 45] as do all living organisms. Additionally, we do not explicitly model the success or failure of beetle mass attack on trees nor the mechanisms that drives the beetle attraction to trees. We assume that there is an initial distribution of beetles in the forest, that is, each tree hosts a certain number of beetles at initial time. The evolution of the beetle populations in each individual tree over time is described by a logistic-type growth that includes the host-tree defensive capabilities against beetles. The later is assumed to be of Holling Type-III functional response (details of the model without feedback of the trees on beetles can be found in [5]).

To model the feedback of tree population on beetle population dynamics, we assume that after the beetles are settled in a host, the capacity of the tree to support beetle population growth depends on the age of the tree. This assumption builds upon some evidences that beetles development after settling in the host correlates with size/age [26, 27, 46–48]. We translate this assumption into the equation modeling the beetle dynamics by setting the carrying capacity *K*_*e*_(*a*) to be dependent on the age of the tree.

Based on biological background, the beetle population grows on a much faster scale than the trees [26, 27, 49–51]. The new beetle adults emerge from last season’s hosts in late summer, and attack new hosts over a 2–3 week period. If the hosts are killed, then the attacking beetles lay eggs that develop through larval stages over the fall, winter and spring [27]. Thus, after the beetles settled into a tree of a certain age the beetle population reaches its steady state before the age of the tree changes. Hence, when modeling the dynamics of the beetle population, the age of trees can be thought of as a parameter. Additionally, we do not structured the beetle population by any individual variability and do not include spatial aspect. Therefore, the rate of change of *B*(*a*, *t*) is only with respect to time *t*, which we write as *dB*(*a*, *t*)/*dt*. That is, the dynamics of the beetle population is described by the ordinary differential equation in (1a). Observe that this equation models the time-evolution of beetles in a single tree, consequently, it does not depend explicitly on *V*. This formulation, which is not standard in age structured Lotka-Mckendrick PDE predator-prey models, constitutes a key ingredient that permits us to simplify the theoretical analysis of both the model and the optimal control problems as well as the numerical simulations.

The rate of change of the population of trees *V*(*a*, *t*) has two terms, the partial derivative with respect to *t* and the partial derivative with respect to *a* (the age of the trees). The PDE is given in (1b). The equations of the full model describing the interaction between dynamics of tree and beetle populations are:

$$\begin{array}{cc} & \frac{dB(a,t)}{dt}=r_{b}B\left(a,t\right)(1-\frac{B(a,t)}{K_{e}\left(a\right)})-\frac{\alpha B{\left(a,t\right)}^{2}}{1+\beta B{\left(a,t\right)}^{2}}. \end{array}$$ (1a)

$$\begin{array}{cc} & \frac{\partial V(a,t)}{\partial t}+\frac{\partial V(a,t)}{\partial a}=-\left[\mu \left(a,t\right)+\mu_{B}\left(a,t\right)+u\left(a,t\right)\right]V\left(a,t\right), \end{array}$$ (1b)

$$\begin{array}{cc} & \mu_{B}\left(a,t\right)=f_{k}\left(a\right)B\left(a,t\right) \end{array}$$ (1c)

$$\begin{array}{cc} & 0\leq t\leq T,\;0\leq a\leq A,\;A,T<\infty \end{array}$$ (1d)

$$\begin{array}{cc} & V\left(a,0\right)=V_{0}\left(a\right), \end{array}$$ (1e)

$$\begin{array}{cc} & V(A,t)\equiv 0, \end{array}$$ (1f)

$$\begin{array}{cc} & V\left(0,t\right)=\int_{0}^{A}b\left(a\right)V\left(a,t\right)da, \end{array}$$ (1g)

$$\begin{array}{cc} & B_{T}\left(a,t\right)=\int_{0}^{A}B\left(a,t\right)\;V\left(a,t\right)\;da. \end{array}$$ (1h)

In the equations, the parameter *A* < ∞ denotes the maximum age of trees and *T* < ∞ the maximum time. The first term in (1a) is a logistic-type growth in which the carrying capacity *K*_*e*_(*a*) depends on the age of the trees. The second term represents the host-tree defensive capabilities against beetles. Note that the state variables *B*(*a*, *t*) defines the average number of beetles per tree of age *a* at time *t*. Hence, the total number of beetles in the forest at a given time is as in (1h). Moreover, the Eq (1a) do not depend explicitly on *V*. The function *μ*(*a*, *t*) is the natural death rate of trees and the function *μ*_*B*_(*a*, *t*) in (1c) is the additional age dependent tree mortality due to the presence of beetles. The function *u*(*a*, *t*) represents the harvest rate of healthy trees, which is the control variable. Eq (1e) specifies the initial age distribution of trees. The Eq (1g) is the boundary condition called the fertility equation, which describes how the replacement of trees occurs in the population. The function *b*(*a*) is the birth rate of trees. In the model (1) the coupling between host trees and beetle population is materialized through the function *μ*_*B*_(*a*, *t*), *K*_*e*_(*a*), and *B*_*T*_. One feedback from the trees to the beetle population occurs via the functional *K*_*e*_(*a*) that defines how the age of trees affects the beetle population temporal evolution in each tree. Another feedback is through the way the total population of beetles is defined in Eq (1h). The feedback from the beetle population to the trees in a stand is modeled through the beetle induced tree mortality function *μ*_*B*_(*a*, *t*) given in (1c), as many of the models of age-structured predator-prey interaction which use the Lotka-Mckendrick equation [30, 32–34]). This function depends on the number of beetles in each tree *B*(*a*, *t*) and on the age-related fraction of trees killed per beetle in a successful attack, *f*_*k*_(*a*).

Throughout the paper, the following assumptions on *b*(*a*), *V*_0_(*a*), *K*_*e*_(*a*), *μ*(*a*, *t*), and *μ*_*B*_(*a*, *t*) are made:

H1 *b* ∈ *L*^∞^(Ω), *b*(*a*, *t*) ≥ 0 *a*.*e*. (*a*, *t*) ∈ Ω.
H2 $\mu \in L_{loc}^{1}\left(\bar{\Omega }\right),\;\mu \left(a,t\right)\geq 0\;a.e.\;\left(a,t\right)\in \Omega ,\;\int_{0}^{A}\mu \left(a,t\right)da=+\infty .$
H3 $\mu_{B}\in L_{loc}^{1}\left(\bar{\Omega }\right),\;\mu_{B}\left(a,t\right)\geq 0\;a.e.\;\left(a,t\right)\in \Omega .$
H4 *V*_0_ ∈ *L*^1^ (0, *A*), *V*_0_(*a*) ≥ 0 *a*.*e*. *a* ∈ (0, *A*).
H5 *K*_*e*_(*a*) ∈ *C*^1^([0, *T*]), *K*_*e*_(*a*) > 0 *a* ∈ [0, *A*],

where Ω = (0, *A*) × (0, *T*) and $\bar{\Omega }=\left[0,A\right]\times \left[0,T\right].$ These assumptions reflect practical observations on biological population and they are needed to prove the theoretical results given in section: “Optimal control existence and characterization”. The assumptions **H1**–**H4** were used in previous works (see e.g., [29]). In section: “Numerical simulations” we specify the functional forms of *b*(*a*), *V*_0_(*a*), *K*_*e*_(*a*), *μ*(*a*, *t*) and *μ*_*B*_(*a*, *t*) used in the specific case studies explored numerically. Furthermore, we choose regions of parameter values for which the model reflects standard harvesting scenarios where, in absence of beetles and harvesting, only a finite amount of trees of a certain age are present at any given time because there is only a finite area available for trees to grow.

## Optimal control problem

In this work, we consider the problem of determining the optimal harvest rate of healthy trees *u*(*a*, *t*), the system *control* variable that maximizes the objective functional (harvest benefit). The harvesting benefit formulation assumes that the benefit obtained from trees is based on the number of trees removed from the forest. Furthermore, the objective functionals do not include discounting future benefits. These formulations are the simplification of reality as most timber is sold based on volume (or biomass amount). In addition, the inclusion of discounting factors is important as they account for present/future money value tradeoffs. However, functionals without discount factors in the context of harvesting can be found in the bibliography (e.g., [30, 52]). Alternative more realistic formulations could be used (e.g., [6, 25, 30, 52, 53]). In this article we use a simplified type of benefit function. Since to the best of our knowledge there is no data available to parameterize the models, our reason for taking such a simplified approach is to keep the mathematical formulations simple to derive theoretical results. The framework can be seen as a tool to initiate the explorations of the implications of the beetle infestation and harvesting disturbance interactions on forest. The insights resulting from this simple framework can then guide the development of data-driven optimal control problems modeling more realistic scenarios. We hope that our simplified formulation provides a foundation for collection of data as well as further insight and analysis by other researchers.

We consider the following three definitions of harvest benefit and corresponding functionals:

1. Total number of trees harvested:
  $$\begin{array}{c} J_{1}\left(u\right)=\int_{0}^{T}\int_{0}^{A}\omega_{1}\;u\left(a,t\right)V\left(a,t\right)\;da\;dt, \end{array}$$ (2)
2. Total amount of wood harvested including trees killed by beetles and other natural causes:
  $$\begin{array}{c} J_{2}\left(u\right)=\int_{0}^{T}\int_{0}^{A}\left[\omega_{1}\;u\left(a,t\right)+\omega_{2}\;\left(\mu_{B}\left(a,t\right)+\mu \left(a,t\right)\right)\right]V\left(a,t\right)\;da\;dt, \end{array}$$ (3)
  and
3. Total benefit:
  $$\begin{array}{ccc} J_{3}\left(u\right) & = & \int_{0}^{T}\int_{0}^{A}[\omega_{1}u\left(a,t\right)+\omega_{2}\left(\mu_{B}\left(a,t\right)+\mu \left(a,t\right)\right)]V\left(a,t\right)\;da\;dt-\\ & & \int_{0}^{T}\int_{0}^{A}\omega_{3}u^{2}\left(a,t\right)\;da\;dt \end{array}$$ (4)
  where *ω*_1_, *ω*_2_, *ω*_3_ > 0 are weights. The product *uV* represents the number of trees harvested while the product (*μ*_*B*_+ *μ*)*V* represents the amount of dead trees collected, which includes those killed by beetles and those that died from natural causes. The term *ω*_3_ *u*^2^ represents the cost of implementing the harvesting and the ecological cost of removing healthy trees from the ecosystem. We assume the units of *ω*_1_ and *ω*_2_ to be dollars per tree and the units of *ω*_3_ to be dollars *per* square tree. Thus, the functionals are expressed in amounts of money (dollars, euros or any other currency). We are assuming that the value of wood is independent of the age of the tree.

The first two objective functionals are used to establish a base of results and the functional (4) has economic relevance as it includes the cost of harvesting trees. The harvesting benefit formulation (2) is the same as the benefit defined in [5] when *ω*_1_ = 1. The underlying assumption is that the total number of trees cut corresponds to the total amount of wood harvested and that the latter is the yield resulting from the forest. This is a very simplified view of the type of benefits that can be provided by the forest. A more realistic scenario, for example, is to suppose that the timber killed by beetles and the trees that die naturally can be, and are often harvested. The harvesting benefit defined in (3) models this situation by incorporating the additional wood provided by dead trees. Observe that if *ω*_1_ = *ω*_2_ in (3), then the revenue associated with the wood is equal across healthy harvested trees, damaged trees (trees killed by beetles), and trees that die naturally. If *ω*_1_ > *ω*_2_, then the harvested wood is more valuable than the wood from trees killed by beetles and other natural causes. An alternative formulation using net benefit and the cost of harvesting is given by (4), where the cost function is quadratic indicating the cost of the intervention is nonlinear. Other functions can be employed but we choose nonlinear cost because it is a common formulation in the context of optimal harvesting problems (see [38, 52, 54] and references therein).

We study three distinct *optimal control problems* consisting of:

P1 the objective functional (2) and system (1);
P2 the objective functional (3) and system (1);
P3 the objective functional (4) and system (1).

More precisely, we maximize each functional (2), (3), and (4) over the set of admissible controls

$$\begin{array}{c} U=\{u\left(a,t\right)\in L^{\infty }\left(\Omega \right):\;0\leq u\left(a,t\right)\leq u_{max}\;a.e.\;\text{in}\;\Omega \} \end{array}$$

for a given set of initial conditions of system (1).

## Optimal control existence and characterization

In this section we first show the existence of solutions along the characteristic for the state Eq (1). After this result has been established, for each of the problems defined in section: “Optimal control problem” we prove the existence of an optimal control and give its characterization.

### Existence and uniqueness of solutions of (1)

Here we discuss the existence and uniqueness of nonnegative solutions of (1). Observe that by construction, the dynamics of beetles in Eq (1a) does not depend explicitly on the variable *V*. Thus, the study of solutions of the system (1) can be performed by first studying the existence and uniqueness of solutions of the initial value problem consisting of (1a) and the initial condition *B*(0, *a*) = *B*_0_(*a*), *B*_0_ > 0. The standard theory of ordinary differential equations guarantees that the age-dependent solution of this initial value problem is nonnegative, unique, and continuous [5].

The analysis in [55, Results 1 and 2] shows that, in certain region of parameters, the Eq (1a) exhibits bi-stability with one local asymptotically stable (LAS) steady state having a lower number of beetles per tree of age *a* that we call *the endemic state*. A LAS steady state with the higher number of beetles per tree of age *a* that we call *the epidemic state*. The steady-state with *B* ≡ 0 that we call *beetle free state* is realized by setting *B*(0, *a*)≡0. Observe that these steady states for the beetle population are functions of age, and we denote them by B*(*a*). Also, by model construction, the total number of beetles in the system is computed using (1h), therefore, if there are no trees in the forest the number of beetles will be identically zero. Furthermore, quantitatively, the endemic and epidemic beetle states in the trees-beetle system differ from the endemic and epidemic states of (1a) (see (1h)).

As discuss in the model formulation section, the age in the Eq (1a) plays the role of a parameter. It was also noticed that the dynamics of the beetle population occurs on a much faster scale that the dynamics of the trees. This implies that, given a certain initial distribution of beetles *B*(0, *a*) = *B*_0_(*a*), the steady-state solutions of the beetle-ODE as a function of the age can be derived by solving (1a). This solutions constitute the age-dependent profile of the steady states solutions B*(*a*). With this fact in mind we next study the existence and uniqueness of the solution of the problem (1b)–(1g) with B*(*a*) a nonnegative unique solution of (1a). That is, we perform the study by setting *μ*_*B*_ = *f*_*k*_(*a*)B*(*a*). In this paper, we consider a solution of (1b)–(1g) as a function *V*(*a*, *t*)∈*L*^∞^(0, *T*; *L*^1^(0, *A*)), absolutely continuous along almost every characteristic line (of equation *a* − *t* = *constant*, with (*a*, *t*)∈[0, *A*] × [0, *T*]) that satisfies (1b)–(1g) a.e. in Ω [29].

Observe that **H1**–**H5** satisfy the assumptions in [29, Theorem 4.1]. Thus, by this theorem the problem (1b)–(1g) has a unique nonnegative solution, which guarantees the existence and uniqueness of the solution of the problem (1) (in the sense described above). To compute this solution along the characteristic lines *a* − *t* = *constant* we define *m*(*a*, *t*) := *μ*(*a*, *t*)+ *μ*_*B*_(*a*, *t*)+ *u*(*a*, *t*) and assume that *B*(*a*, *t*) is known at any given time. This allow us to integrate the solutions along the characteristic lines considering the cases *a* > *t* and *a* < *t* following the approach given, for example, in [56]. The resulting expressions corresponding to the two cases are:

$$\begin{array}{cc} & V\left(a,t\right)=V_{0}\left(a-t\right)\exp(-\int_{0}^{t}m\left(s^{\prime }+a-t,s^{\prime }\right)ds^{\prime }),\;a>s=t. \end{array}$$ (5a)

$$\begin{array}{cc} & V\left(a,t\right)=\int_{0}^{A}b\left(s\right)V\left(s,s+t-a\right)ds\exp(-\int_{0}^{a}m\left(s^{\prime }\right)ds^{\prime }),\;s=a<t. \end{array}$$ (5b)

### Existence and uniqueness of an optimal control

We first study the existence and uniqueness of the two linear optimal control problems **P1** and **P2** define in section: “Optimal control problem”. Additionally, for each of them, we derive the necessary conditions that an optimal solution must satisfy and form the optimality system that consists of the state equations coupled with the adjoint equations and the optimality condition. Furthermore, we provide the characterization of the optimal control. A similar study is performed for the nonlinear optimal control problem **P3**. The proof of the existence of sensitivity, the existence of solutions of the adjoint system as well as the proof of the existence of a unique optimal control for problems **P1** and **P2** follows the result in [29] and are not given in this paper. However, we prove similar results for the nonlinear optimal control problem **P3** in Appendix A: Lipschitz property of the state solution *V*(*a*, *t*) through Appendix E: Uniqueness of the optimal control. All the results are derived considering the state Eqs (1b)–(1g), with B*(*a*) a nonnegative unique solution of (1a).

#### Linear optimal control problems

**Optimal control problem P1**. The result in [29, Chap. 4, Theorem 4.9] guarantees that there exists an optimal control u* maximizing the functional *J*_1_(*u*) over U. The existence of an optimal control provides the basis for the derivation of the necessary conditions for the optimality system. This derivation follows standard methods (see [38] and references therein). Theorem 1 below provides the equations satisfied by the sensitivity *ψ*, Theorem 2 gives the adjoint system of equations for the control problem, and Theorem 3 characterizes the optimal control.

**Theorem 1** Given an optimal control u* and the corresponding state variable V* *the map u* → *V(u) is differentiable in the following sense (Gâteaux sense)*:

$$\begin{array}{c} \lim_{\delta \to 0}\frac{V(u+\delta l)-V(u)}{\delta }\to \psi \end{array}$$

*in L*^∞^(0, *A*) × *L*^∞^(0, *T*), *as δ* → 0, *for* u+δl∈U, *with l* ∈ *L*^∞^(Ω). *The sensitivity equations satisfy*

$$\begin{array}{cc} & L\psi =-lV^{*}, \end{array}$$ (6a)

$$\begin{array}{cc} & \psi \equiv 0\;on\;[0,A]\times \{t=0\}, \end{array}$$ (6b)

$$\begin{array}{cc} & \psi \left(0,t\right)=\int_{0}^{A}b\left(a\right)\psi \left(0,t\right)da\;on\;\left\{a=0\right\}\times \left[0,T\right], \end{array}$$ (6c)

*where* L *is the sensitivity operator given by* $L\psi =\frac{\partial \psi }{\partial t}+\frac{\partial \psi }{\partial a}+(\mu +\mu_{B}+u^{*})\psi$.

The derivation of the adjoint system from the sensitivity equations is carried out using the following relation between the sensitivity operator L and the adjoint operator $L^{*}$:

$$\begin{array}{c} \int_{\Omega }\lambda L\psi \left(a,t\right)da\;dt=\int_{\Omega }\psi \left(a,t\right)L^{*}\lambda da\;dt, \end{array}$$ (7)

where λ is the adjoint variable.

**Theorem 2** *The adjoint system associated with* (6a)–(6c) *satisfies*:

$$\begin{array}{cc} & L^{*}\lambda =\omega_{1}u^{*}, \end{array}$$ (8a)

$$\begin{array}{cc} & \lambda =0\;on\;[0,A]\times \{t=T\}, \end{array}$$ (8b)

$$\begin{array}{cc} & \lambda =0\;on\;\{a=A\}\times [0,T], \end{array}$$ (8c)

where $L^{*}\lambda =-\frac{\partial \lambda }{\partial t}-\frac{\partial \lambda }{\partial a}+(\mu +\mu_{B}+u^{*})\lambda -\lambda \left(0,t\right)b\left(a\right).$ This system has a unique weak solution in *L*^∞^(0, *A*) × *L*^∞^(0, *T*).

The optimality system consists of the state equation coupled with the adjoint equation and the following characterization of the optimal control:

**Theorem 3** *If* $u^{*}\in U$ *is an optimal control maximizing* (2) and V* = *V*(u*) *and* λ *are the corresponding state and adjoint solutions, then the optimal control is given as*

$$\begin{array}{c} u^{*}=\{\begin{array}{ccc} 0 & & if\;\omega_{1}-\lambda <0\\ u_{max} & & if\;\omega_{1}-\lambda >0 \end{array} \end{array}$$ (9)

and u* *is unique*.

**Remark 4** *Observe that μ, μ_B_, b satisfy hypothesis* H5 *in* [29, *Sec. 4.2, p. 164*], *which guarantees that* λ(*a*, *t*) *≠ ω*_1_ *a*.*e*. ∈ Ω, and u* *is a bang-bang control*.

**Optimal control problem P2**. Arguments and methodologies similar to the ones described for optimal control **P1** applied to **P2** guarantee the existence and uniqueness of a *bang-bang control*. Since the state system is the same for both problems, the sensitivity equations associated to the optimal control problem **P2** are as in (6). Consequently, Theorem 1 holds true for problem **P2** too. Similarly, Theorem 2 holds true for problem **P2** as well when Eq (8a) is replaced by

$$L^{*}\lambda =\omega_{1}u^{*}+\omega_{2}\left(\mu +\mu_{B}\right).$$

The characterization of the *bang-bang control* u* for problem **P2** is the same as in Eq (9).

#### Nonlinear optimal control problem P3

The existence of a unique optimal control for **P3** is established using Ekeland’s Principle [30, 31, 57]. However, before we apply this principle several intermediate results are needed. The first of them is the result that gives the Lipschitz property of the solution *V*(*a*, *t*) of (1a) and (1b). This property is proved in Appendix A: Lipschitz property of the state solution *V*(*a*, *t*) and it is used to establish the existence of the sensitivity equations, which we address next, as well as the existence and uniqueness of optimal control. Note that an optimal system that consists of the state and the adjoint problems.

**Optimality conditions**. In order to derive the necessary conditions that an optimal solution must satisfy and to form an optimal system, the objective function *J*_3_(*u*) is differentiated with respect to the control. In this process, since the objective function also depends on the state *V*, the state variable *V* is differentiate with respect to the control *u* in the direction *l*. This is achieved by forming an appropriate difference quotient and pass to the limit. The derivative of the map u∈U→V(u) is called sensitivity. The Theorem 1 provides the differentiability of this state-to-control map as well as the sensitivity equations. We proceed by forming the adjoint system.

The adjoint operator (L) associated with Eq (6) is obtained using the relation (7). Briefly, the process consists in combining (7) with Eq (6a), the initial and boundary conditions (Eqs (6b) and (6c) respectively), and integration by parts to send the derivatives on the differential operator in the sensitivity function *ψ* onto the derivatives of the differential operator in the adjoint system λ. Additionally, the following transversality conditions associated with the adjoint variable λ are applied:

λ=0on[0,A]×{t=T},λ=0on{a=A}×[0,T].

The resulting adjoint system is

$$\begin{array}{cc} & L^{*}\lambda =\omega_{1}u^{*}+\omega_{2}\left(\mu +\mu_{B}\right), \end{array}$$ (10a)

$$\begin{array}{cc} & \lambda =0\;\text{on}\;[0,A]\times \{t=T\}, \end{array}$$ (10b)

$$\begin{array}{cc} & \lambda =0\;\text{on}\;\{a=A\}\times [0,T], \end{array}$$ (10c)

where $L^{*}\lambda =-\frac{\partial \lambda }{\partial t}-\frac{\partial \lambda }{\partial a}+(\mu +\mu_{B}+u^{*})\lambda -\lambda \left(0,t\right)b\left(a\right).$ The right-hand side of (10a) is obtained by differentiating the objective functional (4) with respect to the state variable *V*.

We proceed by stating Theorem 5 concerning the existence of weak solutions of the adjoint system. The proof is given in Appendix B: Optimality conditions.

**Theorem 5** *The weak solution of the adjoint system* (10) *satisfies*

$$\int_{\Omega }\lambda \alpha -z(\omega_{1}u+\omega_{2}\left(\mu +\mu_{b}\right))da\;dt=0$$

*for α in L*^∞^(Ω) *where z satisfies the system*:

$$\begin{array}{l} \frac{\partial z}{\partial a}+\frac{\partial z}{\partial t}+\left(\mu +\mu_{B}+u\right)z=\alpha \;in\;\Omega \\ z\left(a,0\right)=0\;in\;\left[0,A\right]\times \left\{t=0\right\}\\ z\left(0,t\right)=\int_{0}^{A}b\left(a\right)z\left(a,t\right)da\;in\;\left\{a=0\right\}\times \left[0,T\right]. \end{array}$$

**Characterization of the optimal control**. The characterization of the optimal control is given in Theorem 6 and the proof is presented in Appendix C: Characterization of the optimal control. In order to provide this characterization we embed the functional (4) in the space *L*^1^(Ω) by defining [30, 31, 58]

$$\begin{array}{c} J_{3}\left(u\right)=\{\begin{array}{ccc} J_{3}\left(u\right) & & \text{if}\;u\in U\\ +\infty & & \text{if}\;u\notin U. \end{array} \end{array}$$ (11)

Then, we differentiate *J*_3_(*u*) with respect to the control *u*. Observe that *J*_3_(*u*) is a function of the state variable *V*. Thus, we need to differentiate the state variable with respect to the control too.

**Theorem 6** If u* *is an optimal control maximizing* (11) and the corresponding state and adjoint solutions are V* = *V*(u*) *and* λ, *respectively, then*

$$\begin{array}{c} u^{*}=\{\begin{array}{ccc} 0 & & if\;u_{s}<0\\ u_{s} & & if\;0\leq u_{s}\leq u_{max}\\ u_{max} & & if\;u_{s}>u_{max}, \end{array} \end{array}$$ (12)

*where* $u_{s}:=\frac{\left(\omega_{1}-\lambda \right)V^{*}}{2\omega_{3}}$.

**Existence of an optimal control**. In Appendix D: Existence of an optimal control we prove the following result on the existence of optimal control.

**Theorem 7 (Existence of an optimal control)**. *If u^δ^ is an optimal control maximizer of the functional* $J_{3}^{\delta }\left(u\right)$ *then*

$$\begin{array}{c} u^{\delta }=\{\begin{array}{ccc} 0 & & if\;u_{s}^{\delta }<0\\ u_{s}^{\delta } & & if\;0\leq u_{s}^{\delta }\leq u_{max}\\ u_{max} & & if\;u_{s}^{\delta }>u_{max} \end{array} \end{array}$$

*where* $u_{s}^{\delta }:=\frac{\left(\omega_{1}-\lambda \right)V^{*}}{2\omega_{3}}+\frac{\sqrt{\delta }{\hat{\theta }}^{\delta }}{2\omega_{3}}=u_{s}+\frac{\sqrt{\delta }{\hat{\theta }}^{\delta }}{2\omega_{3}},$ *with the function* ${\hat{\theta }}^{\delta }\;\in L^{\infty }\left(\Omega \right)$ *such that* ${\hat{\theta }}^{\delta }=\left|l\right|/l$ *and* $|{\hat{\theta }}^{\delta }|=1$.

**Uniqueness of the optimal control**. The following theorem gives the uniqueness of the optimal control u* maximizer of the functional $J_{3}\left(u\right)$ and its proof is given in Appendix E: Uniqueness of the optimal control.

**Theorem 8 (Uniqueness of the optimal control)**. *If* $\frac{V_{G}}{2\omega_{3}}$ is sufficiently small, there exists one and only one optimal control u* *in* U *minimizing* $J_{3}\left(u\right)$.

## Numerical simulations

Note that when introducing the model we did not specify the trees and beetles species. Additionally, we only give the general properties of the functions representing the carrying capacity (*K*_*e*_(*a*)), the mortality (*μ*_*B*_(*a*, *t*), *μ*(*a*)), the birth rate of trees (*b*(*a*)), and the initial age profile of trees (*V*_0_(*a*)). But in order to do numerical simulations, in section: “Specific form of the functions *K_e_*(*a*); *μ_B_*(*a*, *t*), *μ*(*a*), *b*(*a*), *V*_0_(*a*)”, we give particular forms for these functions based on empirical observations. However, the parameters have not been estimated using real data. Thus, our model is still generic and the forms of the functions we use can be replaced to reflect specific plant/tree-insect systems in situations where data is available and/or educated process-based formulations can be designed.

Also, we assume that there are no trees of maximum age in the population, that is, we assume *V*(*A*, *t*)≡0 for all *t* ∈ [0, *T*] (see (1f)). Hence, in the numerical simulation the number of trees at *a* = *A* is not computed (see section: “Numerical methods”). Additionally, we choose value of parameter for which, in absence of beetles and harvesting, the number of trees of any age is finite. This procedure guarantee that our results correspond to realistic situation: in practice there is only a finite area where only a finite number of trees can grow.

### Specific form of the functions *K*_*e*_(*a*), *μ*_*B*_(*a*, *t*), *μ*(*a*), *b*(*a*), *V*_0_(*a*)

**Fig 1:**
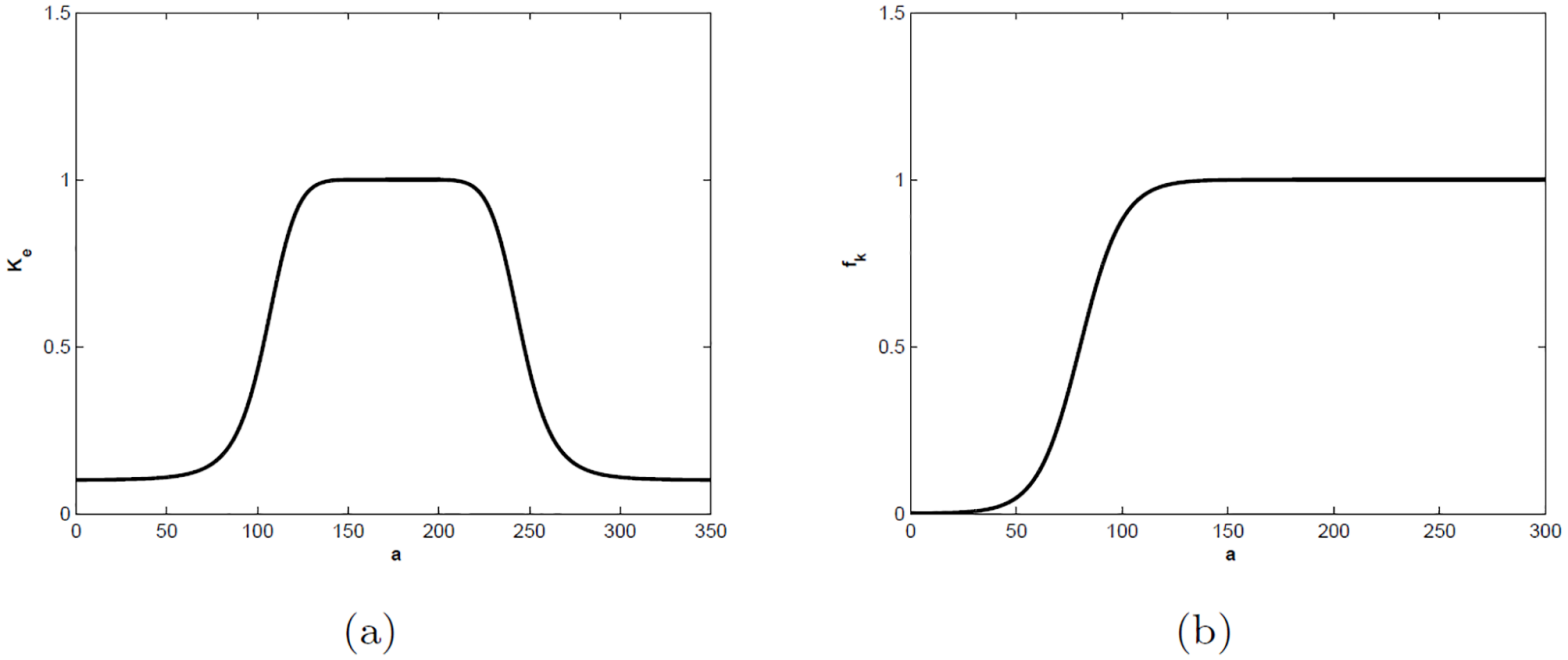
(a): The graph of the carrying capacity of beetles as a function of the age of trees *K*_*e*_(*a*) (*c*_1_ = 175, *c*_2_ = 4, *c*_3_ = .9, *c*_4_ = .1, *s* = 70). (b): The graph of the age-related distribution of the fraction of trees killed per beetle in a successful attack *f*_*k*_(*a*) (*b*_1_ = 80, *b*_2_ = 10).

In order to keep the text simple we do not write the intervals of definition for the functions given in this section but by model construction (see (1d)) they are all assumed to be supported on $\bar{\Omega }=\left[0,A\right]\times \left[0,T\right].$ We make the following assumptions:

1. the carrying capacity of the beetles in trees, which is dependent on the age of trees (*a*), is given by the following generalized bell function
  $$\begin{array}{c} K_{e}\left(a\right)=\frac{c_{3}}{1+{\left|\frac{a-c_{1}}{s}\right|}^{2c_{1}}+c_{4}} \end{array}$$
  and is depicted in Fig 1(a). The biological assumption is that young and old trees have less resources available to support beetle population than mature trees [26, 27, 46–48].
2. The tree defensive rate *α* is assumed to be independent of age. The model can easily be extended to consider a dependence of this parameter on the age of trees. However, since there are no data available to construct the relational function *α*(*a*) we opted to keep the parameter constant for simplicity.
3. As discussed in model formulation (section: Beetle infestation in an age-structured tree population model) we assumed that beetles grow much faster than trees. Hence, the additional mortality due to the presence of beetles, *μ*_*B*_(*a*), depends on the age of the tree as follows:
  $$\begin{array}{c} \mu_{B}\left(a\right)=f_{k}\left(a\right)B^{*}\left(a\right),\;\text{with}\;f_{k}\left(a\right)=\frac{1}{1+\exp(-\frac{a-b_{1}}{b_{2}})} \end{array}$$ (13)
  where B*(*a*) is the steady state solution of beetles of a tree of age *a*. The functional *f*_*k*_(*a*) gives the age-related distribution of the fraction of trees killed per beetle in a successful attack. The choice of a logistic type function (see Fig 1(b) aims to capture the reported interaction between beetle and trees: “Stand conditions and site factors contributing to outbreaks vary somewhat with beetle species and their interactions with their hosts; but almost always require stands of old, larger diameter, less vigorous trees” [59].
4. Following [5] the natural death rate of trees, *μ*(*a*), the birth rate for trees *b*(*a*), and the initial condition (1e) (that is, *V*_0_) are assumed, respectively, as follows
  $$\begin{array}{ccc} & & \mu (a)=A\exp(-a/A)/(A-a), \end{array}$$ (14)
  $$\begin{array}{ccc} & & b\left(a\right)=b_{0}a^{2}\left(A-a\right)\exp\left(-ca\right),\;\text{with}\;b_{0}>0, \end{array}$$ (15)
  $$\begin{array}{ccc} & & V\left(a,0\right)=V_{0}\left(a\right)=v_{0}\exp(-\frac{{\left(a-a_{1}\right)}^{2}}{2s_{1}^{2}}). \end{array}$$ (16)

### Model parameters

The values of the parameters for the B-ODE were chosen so that bi-stability is present in the beetle system and we use the results in [55] as a guide. The values used in the tree Eqs (1b)–(1g) were chosen to guarantee that the forest infested with beetles has a net healthy state, that is, the number of susceptible trees is growing as time evolves. The precise values of the distinct parameters used in the simulations are given in Table 1. For the parameters we found in the literature we indicate the bibliographic reference. Note that some of these values correspond to specific beetles and trees species. However, the calculations we present still hold for generic species since several functions in the model have not been estimated using real data.

**Table 1:** Parameters and values used in the model and numerical simulations. A few bibliographic references are used [55] (denoted by ⋆), [60] (denoted by ⋆⋆), and [48] (denoted by ⋆⋆⋆).

Par.	Value (ref.)	Unity	Description
*α*	0.04086⋆	tree × beetles × year^−1^	Tree defensive rate
*β*	4.102 × 10^−5^⋆	(beetles/tree)^−2^	Reciprocal of the scale of beetles density at which the tree defense saturates
*ω*_1_, *ω*_2_	varied	dollars/tree	Harvesting price weights
*ω*_3_	varied	dollars/(tree)^2^	Harvesting cost weight
*A*	350	years	Maximum age of trees
*a*_1_	75	years	Average age of trees at initial time
*b*_0_	4.65 × 10^−4^	trees × (years)^−4^	Parameter on the fertility funtion
*b*_1_, *b*_2_	80, 10		Parameters on the age-related distribution of trees killed per beetle
*c*	0.1	(years)^−1^	Reciprocal of the time constant in the birth rate functional
*c*_3_	0.9	Beetles	Parameter on age-dependent carrying capacity, *K*_*e*_(*a*)
*c*_1_, *c*_2_, *c*_4_	175, 4, 0.1		Params. on *K*_*e*_(*a*)
*r*_*b*_	2.7⋆⋆	(years)^−1^	Beetle intrinsic reproductive rate
*s*	70⋆⋆⋆	years	Standard deviation for *K*_*e*_(*a*)
*s*_1_	50	years	Standard deviation of the initial age distribution of trees *V*_0_(*a*)
*T*	350	years	Maximum time
*u*_*max*_		(years)^−1^	Maximum harvesting effort
*v*_0_	50	trees	Number of trees with average age of 75 years in *V*_0_(*a*)

### Numerical methods

It should be noted, that the age-dependent natural death rate *μ*(*a*) is unbounded at *a* = *A*, thus leading to a singularity in the state Eq (5) and in the adjoint Eq (8). However, the singularity in the state Eq (5) can be removed using the transformation given in [61], or by explicitly using that *V*(*A*, *t*)≡0 for all *t* ∈ [0, *T*]. The latter was the approach adopted in this paper (see (1f)). Therefore, in the numerical simulation the number of trees at maximum age *a* = *A* is not computed. For the adjoint Eq (8), the same is true except when *ω*_2_ ≠ 0. But along the characteristics, the adjoint equation is of the form studied in [62]. This paper shows the existence and uniqueness of solutions of this type of problems as well as the convergence of the implicit Euler method for obtaining the numerical solutions. On the other hand, since (8a) is only singular at *a* = *A*, the semi-implicit method of [29] also avoids the problem with the singularity and can be applied to find the numerical solution of (8). Both methods were implemented for both equations, the solutions obtained were compared and the rate of convergence as the size of the grid goes to zero was also estimated for different situations. It was found that the relative difference using a 801 × 801 grid was less than 0.001. And since the implicit method requires more iterations, most of the calculations were only done using the semi-implicit method.

The system of Eq (1) has a discontinuity at the origin, that is, when age and time are equal simultaneously zero. The discontinuity propagates along the characteristics [37] and [29, Chap. 4]. Since the numerical methods used integrate along the characteristics there is no loss of accuracy, [29, Chap. 4] and [36]. The following algorithm was used:

1. Construct a grid with the time step equal to the age step
2. Make an initial guess for the optimal control u*
3. Solve the state Eq (5) forward in time and in age
4. Solve the adjoint Eq (8) backward in time and age
5. Update the control u* using either Eqs (9) or (12)
6. Test for convergence, if the convergence criteria is satisfied stop, else go to 2.
7. Calculate the benefit (2), (3) and (4)

Following [29], the state equation for the tree population is discretized using the semi-implicit discretization approach, stated as

$$V\left(i,j\right)=V\left(i-1,j-1\right)/[1+h\left(\mu \left(i\right)+\mu_{b}\left(i,j\right)\right)],$$

where the index *i* refers to the age (*a*) and the index *j* to time (*t*) and *h* is the common discretization step.

The discretization for the adjoint equation (with *ω*_1_ = 1 for the control problem **P1** and *ω*_1_ = 1 and *ω*_2_ = 0 for the optimal control problem **P2**), which is solved back in time and age, is

$$\lambda \left(i-1,j-1\right)=\left(\lambda \left(i,j\right)-temp\right)/[1-h\left(\mu \left(i-1\right)+\mu_{b}\left(i-1,j-1\right)\right)],$$

where

$$temp=h[u^{*}\left(i,j\right)\left(1-\lambda \right)+\omega_{2}\left(\mu \left(i\right)+\mu_{b}\left(i,j\right)\right)+\beta \left(i\right)\lambda \left(1,j\right)].$$

For problems **P1** and **P2**, the optimal control u*(*i*, *j*) = *u*_*max*_ if (1 − λ(*i*, *j*)) is non-negative and u*(*i*, *j*) = 0 otherwise. For problem **P3** the optimal control is given by u* = min{*u*_*max*_, max{*V*(*i*, *j*)(1 − λ(*i*, *j*))/(2*ω*_3_), 0}}.

The integrals in the benefits functions (2), (3) and (4) are calculated using the trapezoidal rule.

## Numerical results

**Fig 2:**
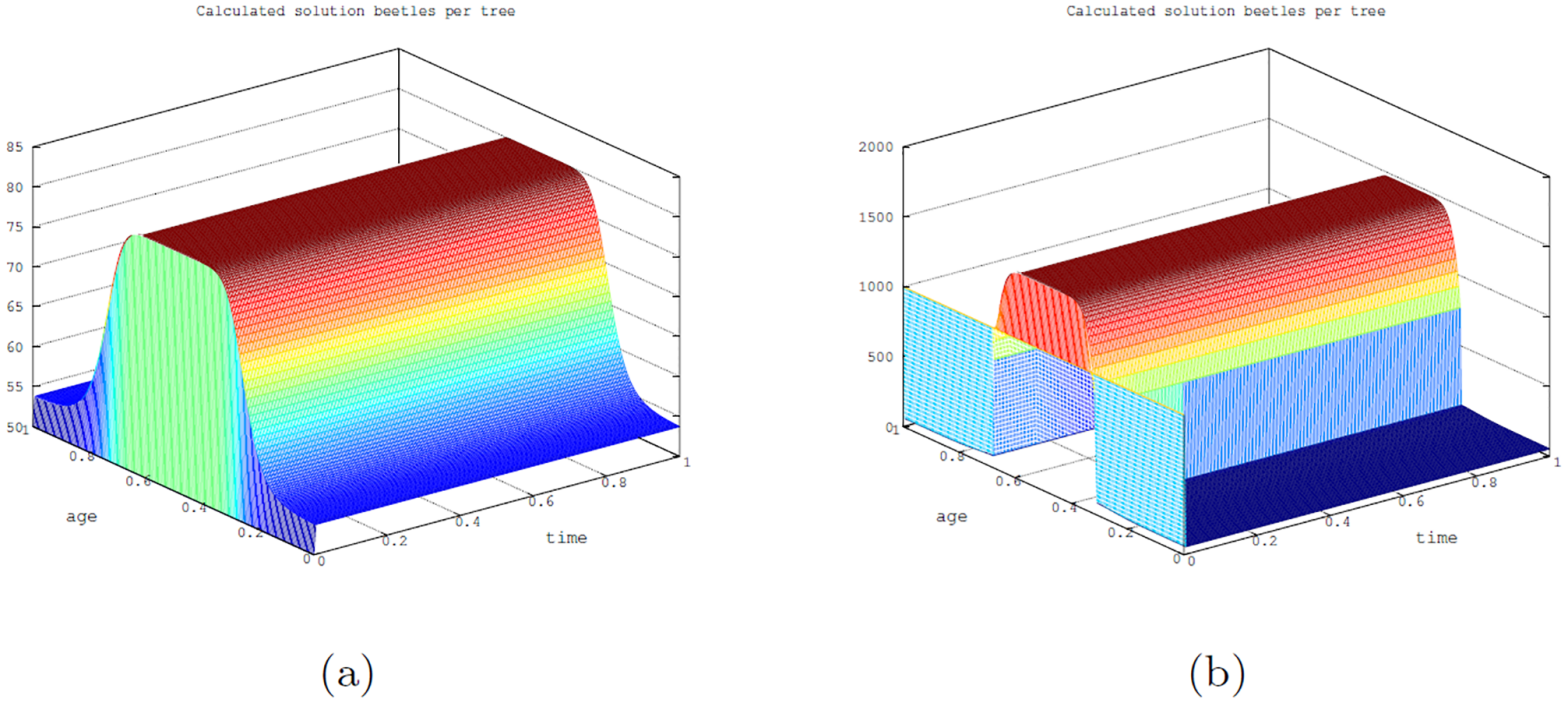
Simulations of model (1) for number of beetles per tree when time (*t*) and age (*a*) are scaled to [0, 1] (where age (*a*) and time (*t*) denote the dimensionless age and time, respectively, ranging from [0, 1] with *a*, *t* = 1 corresponds to 350 years). (a): The number of beetles at the endemic state and (b): The number of beetles at the epidemic state.

First, we show the calculations of the number of beetles per tree for each tree age (*a*) and for all time (*t*). Note that the beetles reach their steady state in a very short time and also since the carrying capacity of the beetles depends on the age of the tree, there are ages of young and old trees for which the epidemic state is the same as the endemic state (see Fig 2).

**Fig 3:**
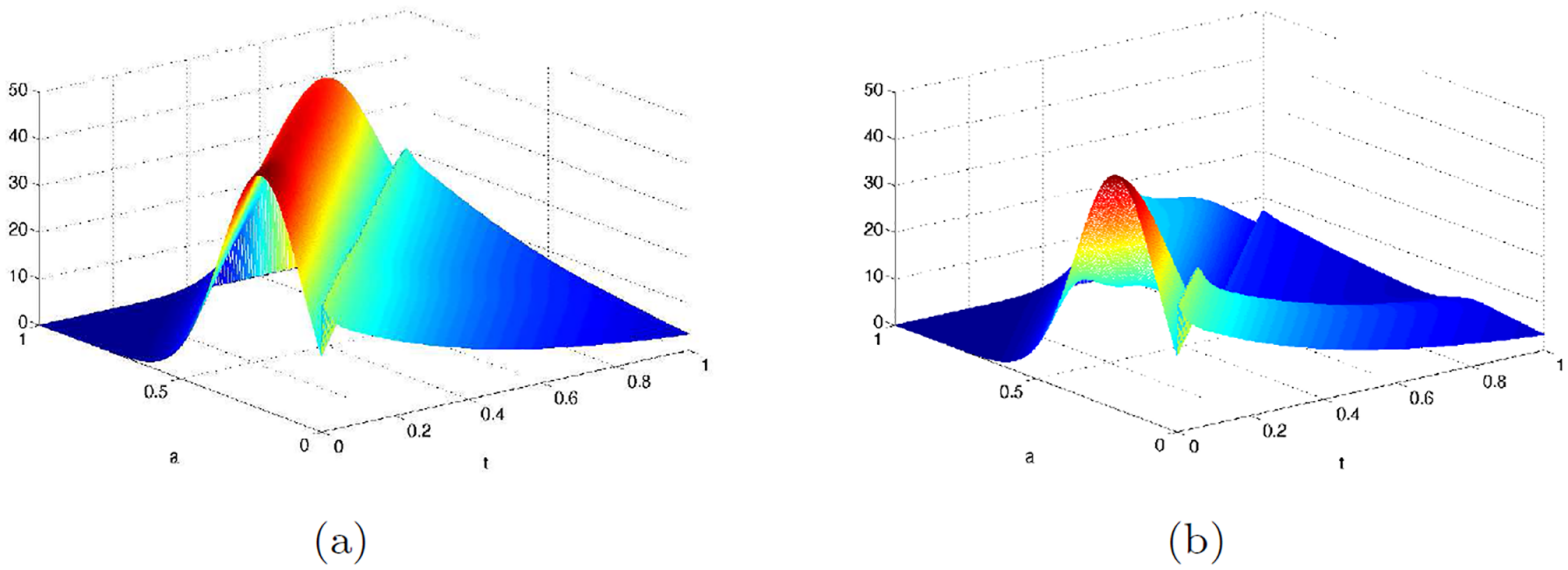
Simulations of model (1) with time (*t*) and age (*a*) scaled to [0, 1]. In the graphs, age (*a*), and time (*t*) denote the dimensionless age and time, respectively, ranging from [0, 1] with *a*, *t* = 1 corresponding to 350 years. (a): Scaled age-structured profile of trees when endemic beetles’ state occur. (b): Scaled age-structured profile of trees when epidemic state of beetles’ state occur.

Simulations with no harvesting are depicted in Fig 3 with the tree age-structured temporal profile for two particular beetles dynamics scenarios: one corresponding to the beetles endemic state and the other to the beetles epidemic state (Fig 3(a) and 3(b), respectively) when time (*t*) and age (*a*) are scaled to [0, 1], which correspond to the dimensional range *t*, *a* ∈ [0, 350] × [0, 350] years.

For simplicity, we set *ω*_1_ = 1, in the benefit functions (2), (3) and (4), and normalized *ω*_2_ and *ω*_3_ with respect to *ω*_1_. If the benefit has *ω*_1_ ≠ 1, the benefit function can be multiplied by the actual price per tree.

**Fig 4:**
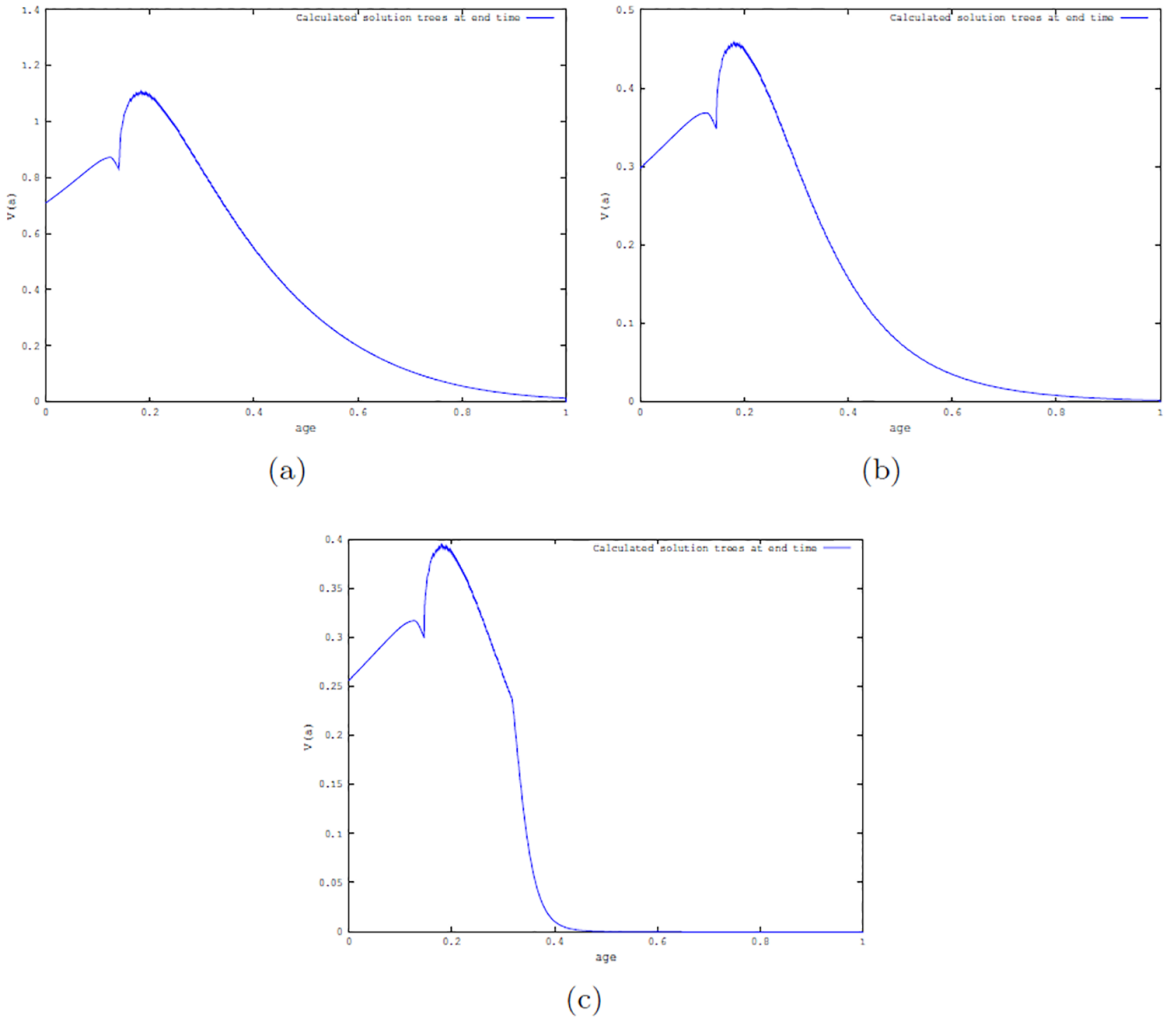
Number of trees at the end time *t* = 1 (i.e., at 350 years) obtained by simulation of optimal control problem P1 with *u*_*max*_ = 10 and *ω*_1_ = 1. (a): When there are no beetles; (b): When beetles are in endemic state; and (c): When beetles are in epidemic states.

**Simulations for optimal control problem P1**. First, we set the maximum harvesting effort *u*_*max*_ = 10, which is the value used in [5]. Fig 4 shows the simulated distribution of the tree population *V*(*a*, *t*) at the end time when there are no beetles present, when the beetles are in the endemic state and the epidemic state.

The values of the benefit function with *ω*_1_ = 1, for the scenarios involving no beetle, beetles in endemic and epidemic states are 25.751, 19.919 and 14.747, respectively. As expected, the benefit is largest when there are no beetles present; this is followed by the case when fewer trees are killed by the beetles.

**Fig 5:**
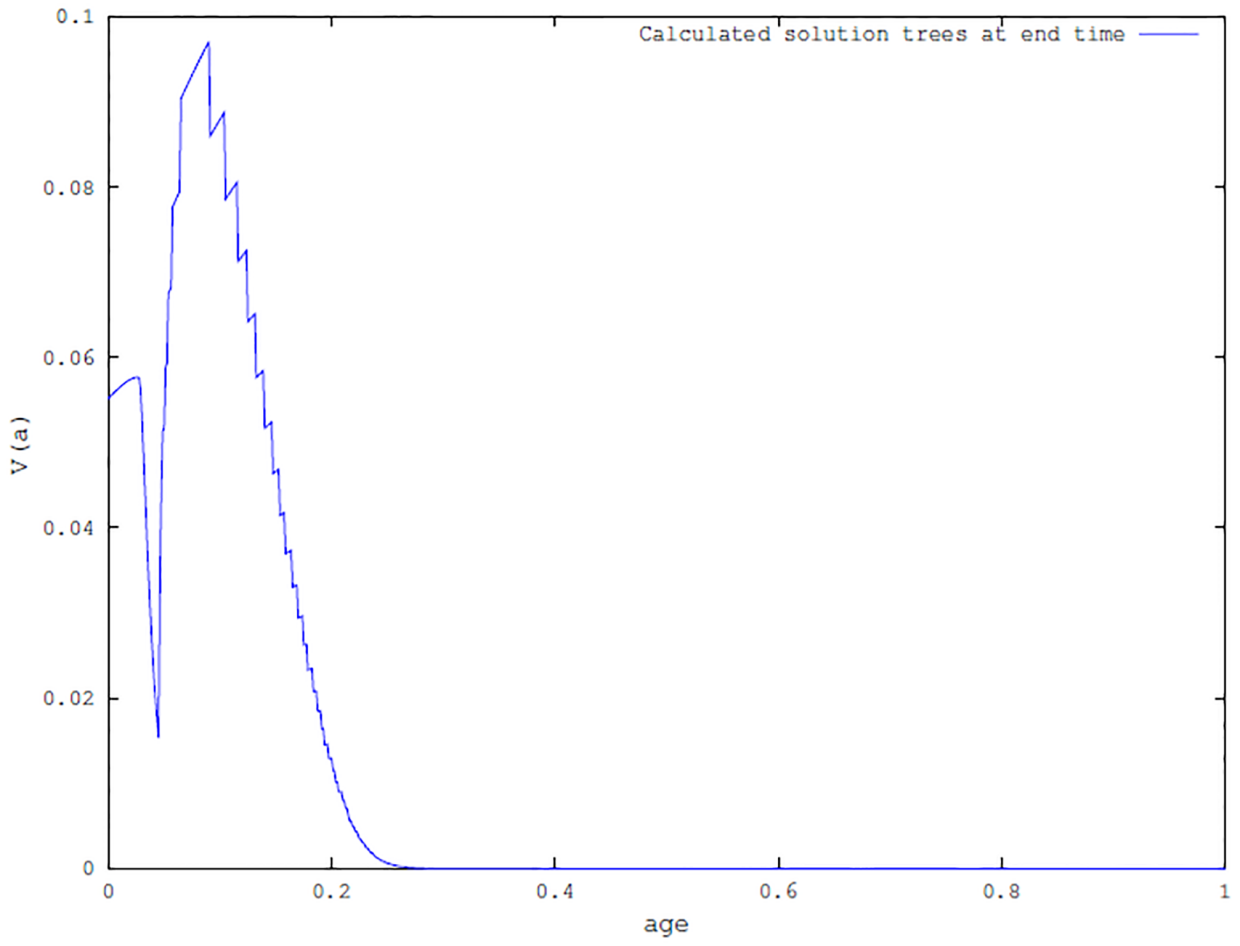
Number of trees at the end time *t* = 1 (corresponding to 350 years) when beetles are in the epidemic state. Results from simulation of the optimal control problem **P1** with *u*_*max*_ = 100 and *ω*_1_ = 1.

To see the effect of changing the maximum harvesting effort (*u*_*max*_) when the beetles are in the epidemic state, the harvesting effort was varied by setting *u*_*max*_ = 20, 50, 100. The corresponding harvesting benefits were 17.801, 20.226 and 21.301. As the harvesting effort increases, the benefit increases slowly. For the no beetle and beetles endemic states, when *u*_*max*_ = 100, the benefits are 26.368 and 22.472, respectively. Fig 5 shows the number of trees at the end time for the epidemic state when *u*_*max*_ = 100. The shape of the optimal control profile is similar to the ones obtained when *u*_*max*_ = 10 for the three beetle scenarios (see S1 File Fig B).

**Fig 6:**
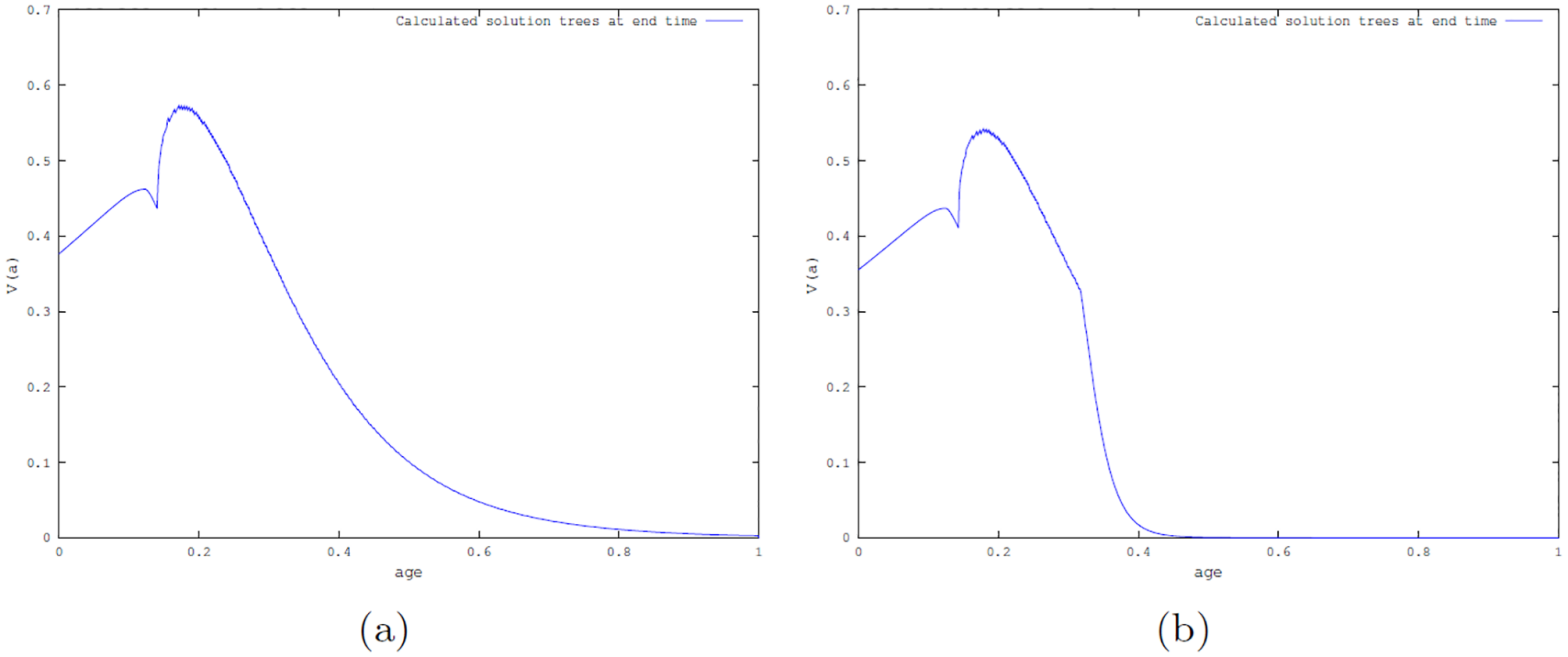
Simulation of the optimal control P2 using *ω*_1_ = 1, *ω*_2_ = .5 and *u*_*max*_ = 10. Number of trees at the end time *t* = 1 (corresponding to 350 years). (a): when beetle population is at the endemic state; (b): when beetle population is at the epidemic state.

**Simulations for optimal control P2**. Calculations were made for the three beetle scenarios setting *ω*_1_ = 1, *ω*_2_ = .5 (i.e., harvested wood are twice as valuable as wood from trees killed by beetles or by other natural causes) and *ω*_1_ = *ω*_2_ = 1 (i.e., all wood are equally valuable). Simulations were implemented using *u*_*max*_ = 10. Fig 6 shows the number of trees as a function of age (*a*) at the end time for the endemic and epidemic cases for the first assumption. Similar graphs for the case *ω*_1_ = *ω*_2_ = 1 are shown in S1 File Fig B.

**Fig 7:**
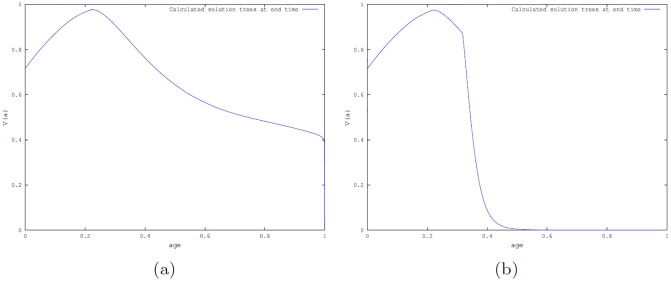
Number of trees at the end time resulting from simulation of the optimal control P3 with *ω*_1_ = 1, *ω*_2_ = .5, *ω*_3_ = .1 and *u*_*max*_ = 10. (a): Beetle population in the endemic state; (b): Beetle population in the epidemic state.

**Fig 8:**
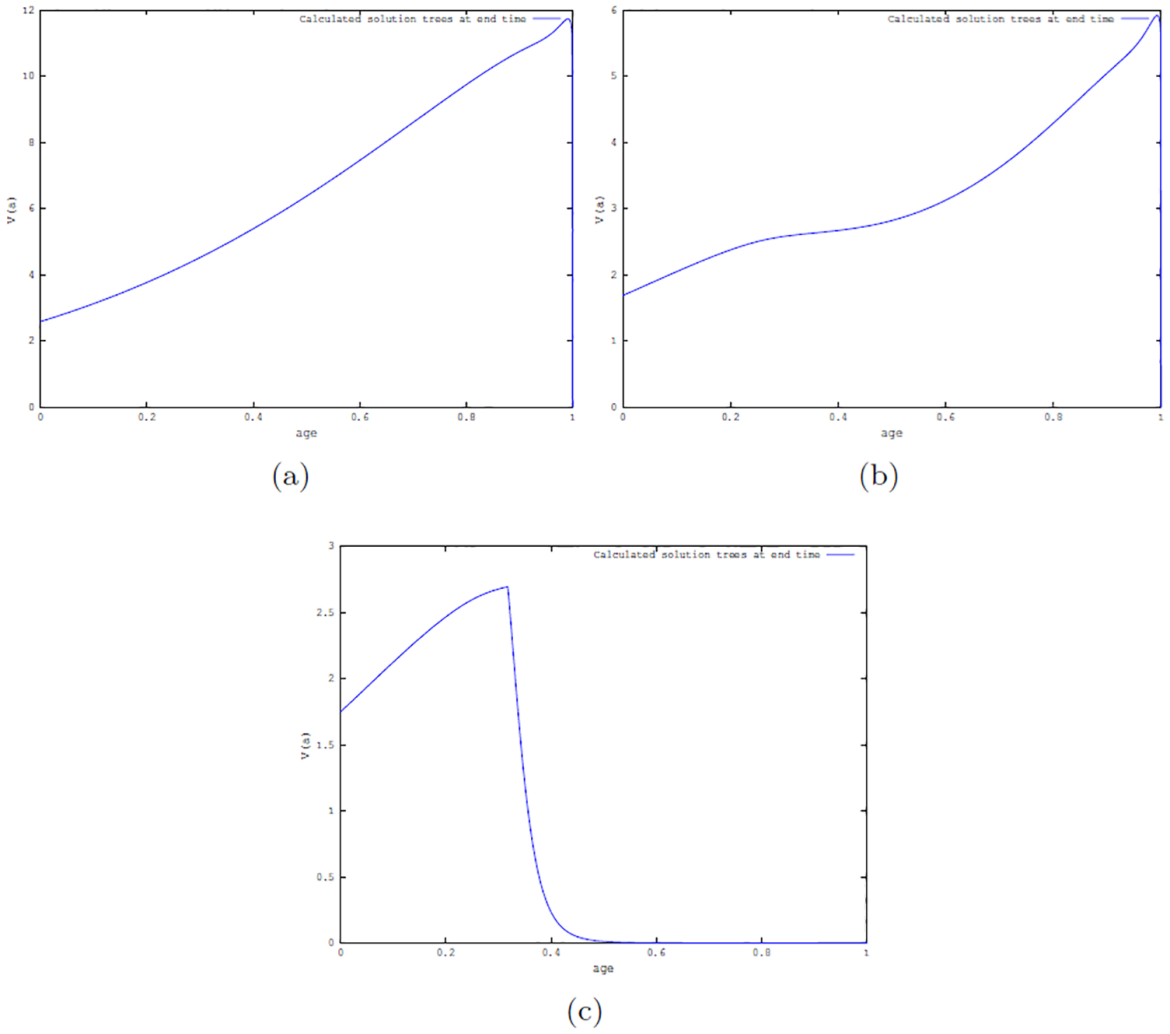
Simulation of the optimal control P3 with *ω*_1_ = 1, *ω*_2_ = .5, *ω*_3_ = 10 and *u*_*max*_ = 10. Number of trees at the end time. (a): in absence of beetles; (b): endemic beetle state; and (c): epidemic beetles states.

**Simulations for optimal control problem P3**. Simulations were implemented with *u*_*max*_ = 10, *ω*_1_ = 1, *ω*_2_ = .5 and varying *ω*_3_ = 0.1, 1, 10, for the three beetle scenarios. Figs 7 and 8 depict the cases *ω*_3_ = 0.1, 10 showing the number of trees as a function age at the end time *t* = 1, i.e, at *a* = 350 years for the scenario when beetle population is either in the endemic state or in the epidemic state.

Table 2 shows the value of the benefits for the three beetles states scenarios with *ω*_3_ = 0.1, 1, 10 and *ω*_1_ = 1, *ω*_2_ = .5. For small and medium values of *ω*_3_ the benefit decreases as the number of beetles increases; however, for larger values of *ω*_3_ the situation is reversed where the benefit increases with increasing beetle population size. This trend is due to the fact that tree harvesting is expensive, resulting in fewer trees been harvested. However, since the trees fell by beetles are also used, we see an increase in the harvesting benefit as the beetles’ states goes from endemic to epidemic states.

**Table 2:** Benefit values for optimal control problem P3 for three distinct harvesting cost weight, *ω*_3_ with fixed *ω*_1_ = 1 and *ω*_2_ = .5.

*ω*_3_	no beetles	endemic state	epidemic state
.1	22.611	19.338	17.687
1	16.392	14.515	14.498
10	8.039	9.654	12.683

Simulations were also carried out using *ω*_1_ = *ω*_2_ = 1 and *ω*_3_ = 10. The values of the benefit are 26.262, 24.964, 25.227, for the no beetles, beetles endemic and epidemic states scenarios respectively. In this case, the benefit is larger for the case of epidemic beetles since there are more trees killed by beetles, which have the same benefit as harvested trees and since there is a cost in harvesting a tree, the number of logged trees is restricted. So in the epidemic case, there are more trees that can be utilized at no harvesting cost.

Simulations of several optimal control functions were performed and graphs of the optimal control functions for problems **P1**, **P2** and **P3** are shown in Fig A in S1 File. Fig B also in S1 File is a simulation of the optimal control problem **P2** using *ω*_1_ = *ω*_2_ = 1 and *u*_*max*_ = 10.

## Discussion of results

Considering the graphs obtained from simulating all the optimal control cases (see Figs 4–8), we observed that at the end time when there are no beetles and when the endemic states are established, there are trees of all ages for all optimal control cases. However, this is not the case for the epidemic state with a fixed maximum harvesting effort *u*_*max*_ (see, Figs 4–8) where there is a greater number of tree deaths and also the absence of older trees in the forest.

Since it as been suggested that old trees provide a greater ecological service than the younger trees [2] our results indicate that the establishment of the epidemic state in the beetle population subject to harvesting would cause a greater ecological damage than the epidemic case. Our study further suggests that, in practice, it may not be necessary to eliminate the beetles but only to keep the population in the endemic state.

Considering the three optimal control problems (**P1**, **P2**, and **P3**), we observed that control problem **P3** which incorporates the cost of harvesting is the most likely to be implemented in practice since there is a cost to harvesting a tree. Usually, this cost is only considered to be economic (manpower, machinery, fuel, etc.) but can also include an ecological component.

## Conclusion

The mathematical framework here presented explicitly links the beetle population dynamics and an age-structured population dynamics subject to tree harvesting stressor. It includes the feedback of beetle population dynamics into the forest dynamics and vice-versa. In this model, we considered trees with age ranging from zero years to a maximum age of 350 years. We studied three scenarios involving the beetle population: no beetles, beetles in endemic and epidemic states, incorporating the effect of the age of the tree on the number of beetles. We used the optimal control to maximize three different benefit functions. As can be expected the harvest benefit is larger when it includes harvesting healthy trees as well as trees killed either by beetles or natural causes; the benefit remains high as long as both types of wood have the same value. This situation can represent, for example, the case in which the wood removed from the forest is used to make paper, biomass or is used in construction, see for example [63] and references therein. Furthermore, the benefit obtained from harvesting the trees and using the dead wood reduces if the value of the wood from death trees is reduced and the cost term in the benefit functional is included. Not surprisingly, as the number of beetles increases, the benefit decreases. This occurs in each case, except when an equal value is placed on the harvested trees and trees killed by beetles. And the benefit functional also includes a cost term (see optimal control problem **P3**). The reason is that, in such situation, the total number of trees killed in the epidemic beetle state is higher in comparison to the mortality of trees in the other two scenarios (no beetles and endemic beetle state). So that the profit generated by the collection of these dead wood surpasses the cost of logging healthy trees.

The models and the simulations are done for a generic model with generic functions and parameters; furthermore, it can be easily adapted to different cases depending on the tree and beetle species. However, in this paper, we have not studied a specific example since we could not find all the necessary data and functions for any one concrete case.

The lack of data sets to validate either our model or more realistic, practical and complex models was the factor that drove the choice of build simplified models. Thus, the simplified mathematical process-based mathematical framework presented and the scenarios explored numerically have more of a theoretical flavor. The work aims to give a better understand of the interactions between harvesting and beetle infestation stressors and their effects on age structure of the forest as well as on the harvesting benefits. The insights derived can guide the development of data-driven optimal control problems modeling the desirable and realistic scenarios. We hope with this first exploration into the beetle-harvesting stressors interaction on the forest dynamics provides a foundation for collection of data, further mathematical modelling and further analysis by other researchers.

## Appendices

### Appendix A: Lipschitz property of the state solution *V*(*a*, *t*)

We state and prove Theorem 9, which provides the Lipschitz property of the solution *V*(*a*, *t*) of (1) in terms of the control *u*(*a*, *t*). This property will be useful to prove the existence of sensitivities as well as existence and uniqueness of optimal control.

**Theorem 9** *The map* u∈U→V *is Lipschitz in the following sense*:

a) $\int_{\Omega }|V-\bar{V}|\;d\Omega \leq K_{A,T}\int_{\Omega }\left|u-\bar{u}\right|\;d\Omega .$
b) ${∥V-\bar{V}∥}_{L^{\infty }\left(0,T;L^{1}\left(0,A\right)\right)}\leq {\hat{K}}_{A,T}{∥u-\bar{u}∥}_{L^{\infty }\left(\Omega \right)}.$

**proof** In order to prove part **a)** we consider the state solution *V*(*a*, *t*) given in (5) and determine the *L*^1^ estimate on Ω. For *t* < *a* < *A* and letting *τ*: = *s*′+ *a* − *t* and *m*(*u*(*a*, *t*)): = *μ*(*a*, *t*)+ *μ*_*B*_(*a*, *t*)+ *u*(*a*, *t*), we have

$$\begin{array}{c} \int_{t}^{A}\left|V-\bar{V}\right|da=\int_{t}^{A}|V_{0}\left(a-t\right)(e^{-\int_{0}^{t}m\left(u\left(\tau ,s^{\prime }\right)\right)ds^{\prime }}-e^{-\int_{0}^{t}m\left(\bar{u}\left(\tau ,s^{\prime }\right)\right)ds^{\prime }})|da \end{array}$$ (17)

this can be re-written as

$$\begin{array}{c} \int_{t}^{A}|V-\bar{V}|da\leq \int_{t}^{A}\left|V_{0}\left(a-t\right)\right|da\times (\int_{t}^{A}|e^{-\int_{0}^{t}m\left(u\left(\tau ,s^{\prime }\right)\right)ds^{\prime }}-e^{-\int_{0}^{t}m\left(\bar{u}\right)\left(\tau ,s^{\prime }\right))ds^{\prime }}da|) \end{array}$$ (18)

Since *V*_0_ satisfies **H4** we have that $\int_{t}^{A}\left|V_{0}\left(a-t\right)\right|da<M_{1},$ where 0 < *M*_1_ < ∞, and inequality (18) can be written as

$$\begin{array}{c} \int_{t}^{A}\left|V-\bar{V}\right|da\leq M_{1}\int_{0}^{A}|e^{-\int_{0}^{t}m\left(u\left(\tau ,s^{\prime }\right)\right)ds^{\prime }}-e^{-\int_{0}^{t}m\left(\bar{u}\left(\tau ,s^{\prime }\right)\right)ds^{\prime }}da|. \end{array}$$ (19)

Since *μ*+ *μ*_*B*_+ *u* satisfies hypothesis **H2** each exponential function inside the integral in (19) is an absolute continuous function. Thus, we can apply the mean value theorem to the derive the inequality

$$\begin{array}{ccc} & & \left|e^{-\int_{0}^{t}m\left(u\left(\tau ,s^{\prime }\right)\right)ds^{\prime }}-e^{-\int_{0}^{t}m\left(\bar{u}\left(\tau ,s^{\prime }\right)\right)ds^{\prime }}|\leq Te^{-\int_{0}^{t}m\left(u_{0}\left(\tau ,s^{\prime }\right)\right)ds^{\prime }}|u-\bar{u}\right|\\ & & \leq TM_{2}\left|u-\bar{u}\right| \end{array}$$ (20)

with $u_{0}\in \left[\bar{u},u\right]$. Substitution of (20) into (19) leads to the follow estimate

$$\begin{array}{c} \int_{t}^{A}|V-\bar{V}|da\leq M_{1}TM_{2}\int_{0}^{A}\left|u-\bar{u}\right|da. \end{array}$$ (21)

Consequently

$$\begin{array}{c} \int_{t}^{A}|V-\bar{V}|da\leq K_{1}\int_{0}^{A}\left|u-\bar{u}\right|da. \end{array}$$ (22)

Next we consider the state solution when *a* < *t* < *T*. Let $\hat{\tau }=s+t-a$ and we proceed to estimate $\int_{0}^{t}\left|V-\bar{V}\right|dt$ as follows:

$$\begin{array}{ccc} & & \int_{0}^{t}\left|V-\bar{V}\right|dt=\int_{0}^{t}|\int_{0}^{A}b\left(s\right)V\left(s,\hat{\tau }\right)dse^{-\int_{0}^{a}m\left(u\left(s^{\prime }\right)\right)ds^{\prime }}-\int_{0}^{A}b\left(s\right)\bar{V}\left(s,\hat{\tau }\right)dse^{-\int_{0}^{a}m\left(\bar{u}\left(s^{\prime }\right)\right)ds^{\prime }}|dt\\ & & =\int_{0}^{t}|\int_{0}^{A}b\left(s\right)[V\left(s,\hat{\tau }\right)-\bar{V}\left(s,\hat{\tau }\right)]ds(e^{-\int_{0}^{a}m\left(u\left(s^{\prime }\right)\right)ds^{\prime }}-e^{-\int_{0}^{a}m\left(\bar{u}\left(s^{\prime }\right)\right)ds^{\prime }})+\\ & & \int_{0}^{A}b\left(s\right)\bar{V}\left(s,\hat{\tau }\right)ds(e^{-\int_{0}^{a}m\left(u\left(s^{\prime }\right)\right)ds^{\prime }}-e^{-\int_{0}^{a}m\left(\bar{u}\left(s^{\prime }\right)\right)ds^{\prime }})+\\ & & \int_{0}^{A}b\left(s\right)[V\left(s,\hat{\tau }\right)-\bar{V}\left(s,\hat{\tau }\right)]dse^{-\int_{0}^{a}m\left(\bar{u}\left(s^{\prime }\right)\right)ds^{\prime }}|dt\\ & & \leq \int_{0}^{t}\int_{0}^{A}|b\left(s\right)[V\left(s,\hat{\tau }\right)-\bar{V}\left(s,\hat{\tau }\right)]|ds|e^{-\int_{0}^{a}m\left(u\left(s^{\prime }\right)\right)ds^{\prime }}-e^{-\int_{0}^{a}m\left(\bar{u}\left(s^{\prime }\right)\right)ds^{\prime }}|dt+\\ & & \int_{0}^{t}\int_{0}^{A}|b\left(s\right)\bar{V}\left(s,\hat{\tau }\right)|ds|e^{-\int_{0}^{a}m\left(u\left(s^{\prime }\right)\right)ds^{\prime }}-e^{-\int_{0}^{a}m\left(\bar{u}\left(s^{\prime }\right)\right)ds^{\prime }}|dt+\\ & & \int_{0}^{t}\int_{0}^{A}|b\left(s\right)[V\left(s,\hat{\tau }\right)-\bar{V}\left(s,\hat{\tau }\right)]|ds\;e^{-\int_{0}^{a}m\left(\bar{u}\left(s^{\prime }\right)\right)ds^{\prime }}dt \end{array}$$ (23)

Now we focus on the two first term on (23). Using an argument similar to the one used when *t* < *a* we can apply the mean value theorem to those two terms, which leads to

$$\begin{array}{ccc} & & \int_{0}^{t}|V-\bar{V}|dt\leq \int_{0}^{t}\int_{0}^{A}|b\left(s\right)[V\left(s,\hat{\tau }\right)-\bar{V}\left(s,\hat{\tau }\right)]|dsN_{1}T\left|u-\bar{u}\right|dt+\\ & & \int_{0}^{t}\int_{0}^{A}|b\left(s\right)\bar{V}\left(s,\hat{\tau }\right)|dsN_{1}T\left|u-\bar{u}\right|dt+\\ & & \int_{0}^{t}\int_{0}^{A}|b\left(s\right)[V\left(s,\hat{\tau }\right)-\bar{V}\left(s,\hat{\tau }\right)]|ds\;e^{-\int_{0}^{a}m\left(\bar{u}\left(s^{\prime }\right)\right)ds^{\prime }}dt \end{array}$$ (24)

Note that by the properties of *V* and *b* it follows that

$$\begin{array}{c} \int_{0}^{A}|b\left(s\right)\bar{V}\left(s,\hat{\tau }\right)|ds;\;\int_{0}^{A}|b\left(s\right)[V\left(s,\hat{\tau }\right)-\bar{V}\left(s,\hat{\tau }\right)]|ds\in L^{\infty }\left(0,A\right). \end{array}$$

Thus, there exists $0\leq N_{2},N_{3}<\infty$ such that

$$\begin{array}{ccc} & & \int_{0}^{t}\left|V-\bar{V}\right|dt\leq \int_{0}^{t}[AN_{2}N_{1}T|u-\bar{u}|+AN_{3}N_{1}T\left|u-\bar{u}\right|]dt+\\ & & \int_{0}^{t}\int_{0}^{A}|b\left(s\right)[V\left(s,\hat{\tau }\right)-\bar{V}\left(s,\hat{\tau }\right)]|ds\;e^{-\int_{0}^{a}m\left(\bar{u}\left(s^{\prime }\right)\right)ds^{\prime }}dt \end{array}$$ (25)

On the other hand since **H1**–**H3** are satisfied there exist 0 ≤ *N*_4_ < ∞ so that the following estimate follows

$$\begin{array}{c} \int_{0}^{A}|b\left(s\right)[V\left(s,\hat{\tau }\right)-\bar{V}\left(s,\hat{\tau }\right)]|ds\;e^{-\int_{0}^{a}m\left(\bar{u}\left(s^{\prime }\right)\right)ds^{\prime }}\leq N_{4}\int_{0}^{A}|V\left(s,\hat{\tau }\right)-\bar{V}\left(s,\hat{\tau }\right)|ds. \end{array}$$ (26)

Substitution of (26) into (25) yields

$$\begin{array}{c} \int_{0}^{t}|V-\bar{V}|dt\leq \int_{0}^{t}A\left(N_{2}+N_{3}\right)N_{1}T\left|u-\bar{u}\right|dt+\int_{0}^{A}N_{4}\int_{0}^{t}|V-\bar{V}|dtds \end{array}$$ (27)

Letting *N*_*AT*_ = *A*(*N*_2_ + *N*_3_)*T* and applying Grönwall’s inequality in integral form to (27) yields:

$$\begin{array}{c} \int_{0}^{t}|V-\bar{V}|dt\leq N_{AT}e^{AN_{4}}\int_{0}^{t}|u-\bar{u}|dt=K_{2}\int_{0}^{t}\left|u-\bar{u}\right|dt. \end{array}$$ (28)

To complete the proof of part a) we combine (22) and (28) to obtain the following estimate

$$\begin{array}{c} \int_{\Omega }|V-\bar{V}|d\Omega =\int_{0}^{A}\int_{0}^{t}|V-\bar{V}|da\;dt+\int_{0}^{A}\int_{t}^{A}|V-\bar{V}|da\;dt\leq K_{A,T}\int_{\Omega }\left|u-\bar{u}\right|da\;dt, \end{array}$$ (29)

where *K*_*A*, *T*_ = *K*_1_ + *K*_2_.

In order to prove part **b)** we find the *L*^∞^ estimates of the state solution by considering the *L*^1^ estimates of $|V-\bar{V}|$ over *a* ∈ [0, *A*]. From (22) and (28) we have

$$\begin{array}{c} \int_{0}^{A}|V-\bar{V}|da\leq K_{1}\int_{0}^{A}|u-\bar{u}|da+K_{2}\int_{0}^{A}\left|u-\bar{u}\right|da \end{array}$$ (30)

Taking the essential supremum overall *a* ∈ [0, *A*] leads to

$$\begin{array}{c} {∥V-\bar{V}∥}_{L^{\infty }\left(0,T;L^{1}\left(0,A\right)\right)}\leq {\hat{K}}_{A,T}∥u-\bar{u}∥{|}_{L^{\infty }\left(\Omega \right)}, \end{array}$$

which completes the proof.

### Appendix B: Optimality conditions

Theorem 1 gives the differentiability of the state-to-control map and its proof is given next.

**Proof of Theorem 1**. By Theorem 9 the map u∈U→V(u) is Lipschitz in *L*^∞^. This result guarantees the existence of the Gâteux derivative *ψ* (see for example [64, p. 17], [31, 65]). Hence, taking the limit of the quotient representations give *ψ*, which satisfies system (6).

We proceed by proving existence of weak solutions of the adjoint system which is given in Theorem 5

**Proof of Theorem 5**. The result follows from the sensitivity and adjoint systems (Eqs (1) and (10) respectively), with *α* = −*lV*. The existence of solution of the adjoint system is established using methods similar to the ones used to prove the existence of solution of (1).

### Appendix C: Characterization of the optimal control

The characterization of the optimal control (u*) is given in Theorem 6. In order to characterize u*, we first embed the functional (4) in the space *L*^1^(Ω) by defining

$$\begin{array}{c} J_{3}\left(u\right)=\{\begin{array}{ccc} J_{3}\left(u\right) & & \text{if}\;u\in U\\ +\infty & & \text{if}\;u\notin U \end{array} \end{array}$$ (31)

and then differentiate $J_{3}\left(u\right)$ with respect to the control *u*. Since $J_{3}\left(u\right)$ is a function of the state variable *V* we need to differentiate the state variable with respect to the control too. The proof is given next.

**Proof of Theorem 6**. Let $u_{s}:=\frac{\left(\omega_{1}-\lambda \right)V^{*}}{2\omega_{3}}$, u+δl∈U for *δ* > 0, and *V*^*δ*^ be the corresponding solution of the state Eq (1b). Note that the linear adjoint equation has a weak solution λ (see Theorem 5). Since we seek to maximize the functional *J*_3_, we compute the directional derivative of *J*_3_ with respect to *u* in the direction *l* at u* as follows:

$$\begin{array}{ccc} & 0 & \geq \lim_{\delta \to 0^{+}}\frac{J_{3}\left(u^{*}+\delta l\right)-J_{3}\left(u^{*}\right)}{\delta }\\ & = & \lim_{\delta \to 0^{+}}\int_{0}^{T}\int_{0}^{A}\{\left[\omega_{1}u^{*}+\omega_{2}\left(\mu +\mu_{B}\right)\right]\frac{V^{\delta }-V^{*}}{\delta }+\omega_{1}lV^{*}-\omega_{3}\left(2lu^{*}+l^{2}\delta \right)\}da\;dt\\ & = & \int_{0}^{T}\int_{0}^{A}\{\psi \left[\omega_{1}u^{*}+\omega_{2}\left(\mu +\mu_{B}\right)\right]+\omega_{1}lV^{*}-2\omega_{3}lu^{*}\}da\;dt\\ & = & \int_{0}^{T}\int_{0}^{A}(\psi L^{*}\lambda +\omega_{1}lV^{*}-2\omega_{3}lu^{*})da\;dt\\ & = & \int_{0}^{T}\int_{0}^{A}(L\psi \lambda +\omega_{1}lV^{*}-2\omega_{3}lu^{*})da\;dt \end{array}$$

in the appropriate weak sense. The sensitivity operator as given in Theorem 1 is used to obtain the following expression

$$\begin{array}{c} 0\geq \int_{0}^{T}\int_{0}^{A}[lV^{*}\left(\omega_{1}-\lambda \right)-2\omega_{3}lu^{*}]da\;dt \end{array}$$ (32)

We let the variation of *l* have support on the set U, where *l* can have arbitrary sign. By standard arguments we obtain the given characterization.

### Appendix D: Existence of an optimal control

The first step to proof the existence and uniqueness of an optimal control for problem **P3** is to guarantee a Lipschitz condition of the adjoint system with respect to the controls. This property is given in Theorem 10 and is established following arguments similar to the ones used in Theorem 9.

**Theorem 10** *The weak solution* λ ∈ *L*^∞^(0, *T*, *L*^1^(0, *A*)) *of adjoint system* (10) *with the control* u∈U *satisfies*

$$\begin{array}{c} {∥\lambda -\bar{\lambda }∥}_{L^{\infty }\left(\Omega \right)}\leq {∥u-\bar{u}∥}_{L^{\infty }\left(\Omega \right)}. \end{array}$$

The functional $J_{3}\left(u\right)$ defined in (31) is lower semicontinuous with respect to strong *L*^1^ convergence. However, it is not lower semicontinous with respect to weak *L*^1^ convergence. Thus, in general it does not attain its infimum on U and the existence of optimal control is not guaranteed. Hence, to overcome this situation we use the Ekeland’s Variational Principle [57, 58] that guarantees the existence of a maximizing sequence satisfying the conditions given next. For *δ* > 0, there exists *u*^*δ*^ ∈ *L*^1^(Ω) such that

1. $J_{3}\left(u^{\delta }\right)>\underset{u\in U}{\inf}J_{3}\left(u\right)+\delta ,$
2. $J_{3}\left(u^{\delta }\right)=\max_{u\in U}J_{3}\left(u\right)-\sqrt{\delta }({∥u^{\delta }-u∥}_{L^{1}\left(\Omega \right)}).$

However, before apply this principle we prove the semicontinuity of the functional with respect to weak *L*^1^ convergence, which will be needed to prove existence of optimal control. After the semicontinuity property is asserted, we show that the maximizer *u*^*δ*^ of the approximate functional converges to the optimal control *u*⋆ ∈ *L*^∞^(Ω).

**Theorem 11 (Lower semicontinuity)**. *The functional* $J_{3}\left(u\right)$ *in* (31) *is lower semicontinuous with respect to weak L*^1^(Ω) *convergence*.

**Proof**. Let *u*^*n*^ → *u* in *L*^1^(Ω). Assume that *V*(*a*, *t*) and *V*^*n*^(*a*, *t*) are the state solutions corresponding to the controls *u*(*a*, *t*) and *u*^*n*^(*a*, *t*), respectively, then Theorem 9 guarantees that

$$\begin{array}{c} V^{n}\to V\;\text{in}\;L^{1}\left(\Omega \right) \end{array}$$

Thus, by [30, Theorem 3.4] on a subsequence we have

$$\begin{array}{c} V^{n}\to V\;\text{and}\;{\left(u^{n}\right)}^{2}\to u^{2}\;\text{a.e.}\;\text{in}\;\Omega . \end{array}$$

Next we consider the linear terms in the functional (4)

$$\begin{array}{ccc} & & \left|\int_{\Omega }\omega_{1}\left(u^{n}V^{n}-uV\right)+\omega_{2}\left(\mu_{B}+\mu \right)\left(V^{n}-V\right)d\Omega \right|\\ & & \leq \int_{\Omega }\omega_{1}|u^{n}-u|V^{n}d\Omega +\int_{\Omega }\omega_{1}|V^{n}-V|u\;d\Omega +\int_{\Omega }\omega_{2}\left(\mu_{B}+\mu \right)\left|V^{n}-V\right|d\Omega \\ & & \leq C_{1}{∥u^{n}-u∥}_{L^{1}\left(\Omega \right)}+C_{2}{∥V^{n}-V∥}_{L^{1}\left(\Omega \right)}\\ & & \leq C{∥u^{n}-u∥}_{L^{1}\left(\Omega \right)}. \end{array}$$

Thus

$$\begin{array}{c} \omega_{1}u^{n}V^{n}+\omega_{2}\left(\mu_{B}+\mu \right)V^{n}\to \omega_{1}uV+\omega_{2}\left(\mu_{B}+\mu \right)V. \end{array}$$

By Fatou’s Lemma [66] we have that on a subsequence

$$\begin{array}{ccc} \int_{\Omega }[\omega_{1}u+\omega_{2}\left(\mu_{B}+\mu \right)]Vd\Omega & = & \int_{\Omega }\underset{n\to \infty }{lim inf}[\omega_{1}u^{n}+\omega_{2}\left(\mu_{B}+\mu \right)]V^{n}d\Omega \\ & \leq & \underset{n\to \infty }{lim inf}\int_{\Omega }[\omega_{1}u^{n}+\omega_{2}\left(\mu_{B}+\mu \right)]V^{n}d\Omega , \end{array}$$ (33)

and

$$\begin{array}{c} \int_{\Omega }\omega_{3}u^{2}d\Omega =\int_{\Omega }\underset{n\to \infty }{lim inf}\omega_{3}{\left(u^{n}\right)}^{2}d\Omega \leq \underset{n\to \infty }{lim inf}\int_{\Omega }\omega_{3}{\left(u^{n}\right)}^{2}d\Omega . \end{array}$$ (34)

Combining (33) and (34) yields

$$\begin{array}{c} J_{3}\left(u\right)=\int_{\Omega }\{[\omega_{1}u+\omega_{2}\left(\mu_{B}+\mu \right)]V-\omega_{3}u^{2}\}d\Omega \leq \underset{n\to \infty }{lim inf}J_{3}\left(u^{n}\right). \end{array}$$

**Existence of an optimal control**:

Let

$$u_{s}^{\delta }:=\frac{\left(\omega_{1}-\lambda \right)V^{*}}{2\omega_{3}}.+\frac{\sqrt{\delta }{\hat{\theta }}^{\delta }}{2\omega_{3}}=u_{s}+\frac{\sqrt{\delta }{\hat{\theta }}^{\delta }}{2\omega_{3}},$$

where the function ${\hat{\theta }}^{\delta }\;\in L^{\infty }\left(\Omega \right)$ such that ${\hat{\theta }}^{\delta }=\left|l\right|/l$ and $|{\hat{\theta }}^{\delta }|=1$.

**Proof of Theorem 7**. Note that *u*^*δ*^ is an optimal control maximizing $J_{3}^{\delta }.$ Therefore, we have

$$\begin{array}{ccc} & 0 & \geq \lim_{a\to 0^{+}}\frac{J_{3}^{\delta }\left(u^{\delta }+al\right)-J_{3}^{\delta }\left(u^{\delta }\right)}{a}\\ & = & \lim_{a\to 0^{+}}\frac{J_{3}\left(u^{\delta }+al\right)-J_{3}\left(u^{\delta }\right)}{a}-\sqrt{\delta }{∥l∥}_{L^{1}\left(\Omega \right)}\\ & = & \int_{0}^{T}\int_{0}^{A}[lV^{\delta }\left(\omega_{1}-\lambda^{\delta }\right)-2\omega_{3}lu^{\delta }+\sqrt{\delta }{\hat{\theta }}^{\delta }]da\;dt \end{array}$$

The last equality follows from (32). Standard optimal control arguments lead to the desired result.

### Appendix E: Uniqueness of the optimal control

**Proof of Theorem 8**. For simplicity of notation we define G:U→U by

$$G\left(u\right):=F(\frac{(\omega_{1}-\lambda )V}{2\omega_{3}}),$$

where

$$\begin{array}{c} F\left(x\right)=\{\begin{array}{ccc} 0 & & if\;x<0\\ x & & if\;0<x<u_{max}\\ u_{max} & & if\;x>u_{max}. \end{array} \end{array}$$

with *V* and λ being the state and adjoint solutions corresponding to *u*. Using the Lipschitz properties of *V* and λ in Theorems 9 and 10, respectively we have

$$\begin{array}{ccc} ∥G\left(u\right)-G\left(\bar{u}\right)∥ & \equiv & {∥F(\frac{(\omega_{1}-\lambda )V}{2\omega_{3}})-F(\frac{\left(\omega_{1}-\bar{\lambda }\right)\bar{V}}{2\omega_{3}})∥}_{L^{\infty }\left(\Omega \right)}\\ & \leq & {∥\frac{(\omega_{1}-\lambda )V}{2\omega_{3}}-\frac{\left(\omega_{1}-\bar{\lambda }\right)\bar{V}}{2\omega_{3}}∥}_{L^{\infty }\left(\Omega \right)}\\ & \leq & \frac{C_{G}}{2\omega_{3}}{∥u-\bar{u}∥}_{L^{\infty }\left(\Omega \right)} \end{array}$$ (35)

Where *C*_*G*_ depends on the *L*^∞^ bounds on the state and adjoint solutions and on the Lipschitz constants.

Note that if $\frac{C_{G}}{2\omega_{3}}<1$ the map *G* has a fixed point u*. In order to show that this fixed point is an optimal control, we use the approximate minimizers *u*^*δ*^ from the Ekeland’s Principle and the corresponding state *V*^*δ*^ and adjoint λ^*δ*^. From Theorem 7 it follows that

$$\begin{array}{c} {∥F(\frac{\left(\omega_{1}-\lambda^{\delta }\right)V^{\delta }}{2\omega_{3}})-F(\frac{\left(\omega_{1}-\lambda^{\delta }\right)V^{\delta }+\sqrt{\delta }{\hat{\theta }}^{\delta }}{2\omega_{3}})∥}_{L^{\infty }\left(\Omega \right)}\leq {∥\frac{\sqrt{\delta }{\hat{\theta }}^{\delta }}{2\omega_{3}}∥}_{L^{\infty }\left(\Omega \right)}=\frac{\sqrt{\delta }}{2\omega_{3}} \end{array}$$ (36)

We proceed by showing that *u*^*δ*^ → u* in *L*^∞^(Ω) using (35) and (36).

$$\begin{array}{ccc} ∥u^{*}-u^{\delta })∥ & \equiv & {∥F(\frac{\left(\omega_{1}-\lambda \right)V^{*}}{2\omega_{3}})-F(\frac{\left(\omega_{1}-\lambda^{\delta }\right)V^{\delta }}{2\omega_{3}})∥}_{L^{\infty }\left(\Omega \right)}\\ \leq \frac{C_{G}}{2\omega_{3}}{∥u^{*}-u^{\delta }∥}_{L^{\infty }\left(\Omega \right)}+\frac{\sqrt{\delta }}{2\omega_{3}} \end{array}$$

Thus, the desired convergence holds. We complete the proof by showing that u* is the minimizer of the functional $J_{3}\left(u\right)$. Combining the fact that $J_{3}$ is lower semicontinous and the Ekeland’s Principle leads to following inequality

$$\begin{array}{c} J_{3}\left(u^{\delta }\right)\leq \underset{u\in U}{\inf}J_{(}u)+\delta \end{array}$$

Since *u*^*δ*^ → u* as *δ* → 0 it follows that

$$\begin{array}{c} J_{3}\left(u^{\delta }\right)\leq \underset{u\in U}{\inf}J_{3}\left(u\right). \end{array}$$

## Supporting information

S1 File Appendix F. Complementary numerical results. Fig A: Optimal Control Functions. Fig B: A simulation of problem **P2**.
(PDF)
