# Supplementary material for: Maximizing tree harvesting benefit from forests under insect infestation disturbances
Source: PLoS One. 2018 Aug 2;13(8):e0200575. doi: 10.1371/journal.pone.0200575 (PMC6071987; doi:10.1371/journal.pone.0200575)
Supplement: S1 File — Appendix F. Complementary numerical results. Fig A: Optimal Control Functions. Fig B: A simulation of problem P2. (PDF) [file pone.0200575.s001.pdf]

Appendix F: Complementary numerical results

Fig A shows the graphs of the optimal control functions for problems **P1**, **P2** and **P3**. Fig A (a) depicts the optimal control for problem **P1** with beetles in the epidemic state and $u_{max} = 100$. All the optimal control involving control functions for problems **P1** and **P2** are bang-bang and they all have similar shapes. Figs A (b) - (d) depict the optimal control for problem **P3** when there are no beetles, beetles in endemic and epidemic states, respectively, with $\omega_1 = \omega_2 = .5$ and $u_{max} = 10$. Fig B depicts simulations for optimal control problem **P2**.

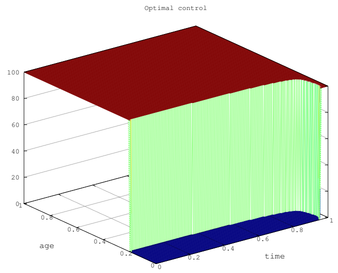

(a)

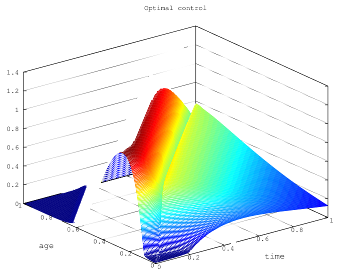

(b)

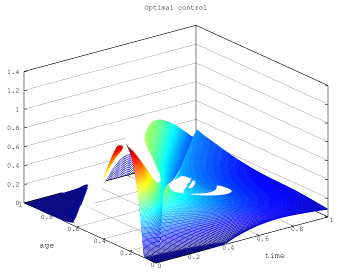

(c)

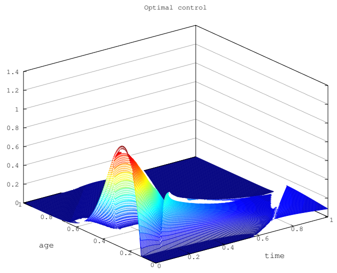

(d)

Fig A. Optimal control functions for the cases of (a): Control problem **P1** with epidemic beetles and $u_{max} = 100$. (b), (c) and (d): Control problem **P3** when there are no beetles, endemic and epidemic states, respectively, with $\omega_1 = \omega_2 = .5$ and $u_{max} = 10$.

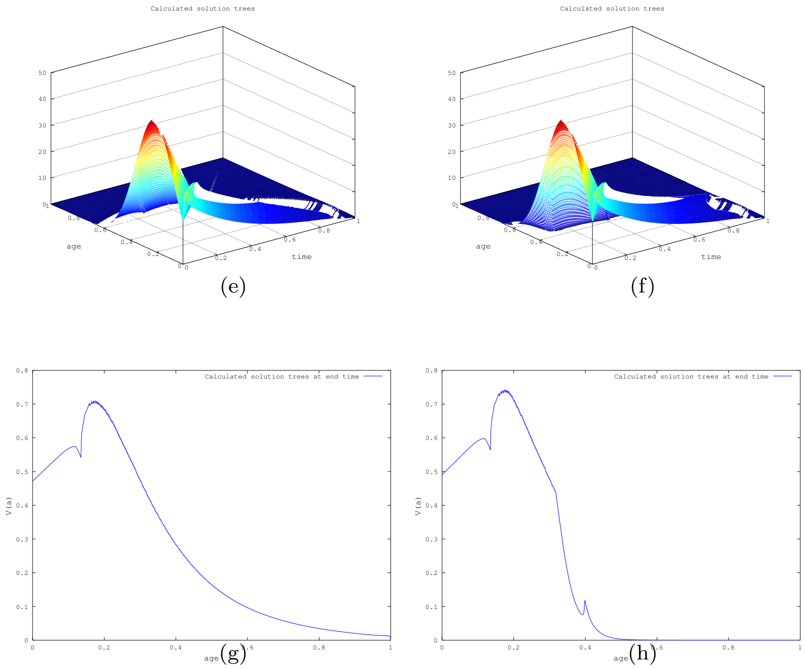

Fig B. Simulation of the optimal control problem **P2** using $\omega_1 = \omega_2 = 1$ and $u_{max} = 10$. (a),(b): Number of trees; and (c),(d): Number of trees at the end time $t = 1$ (corresponding to 350 years). (a),(c): The endemic state; and (b),(d): epidemic states.
